# Cell Membrane- and Vesicle-Based Bionic Nanodrugs: Applications in Central Nervous System Diseases and Exploration of Nasal–Cerebral Delivery

**DOI:** 10.3390/gels11110846

**Published:** 2025-10-22

**Authors:** Fan Ding, Runzhe Hou, Bing Han, Xuexun Fang

**Affiliations:** 1 Key Laboratory for Molecular Enzymology and Engineering, Ministry of Education, School of Life Sciences, Jilin University, Changchun 130012, China; dingfan24@mails.jlu.edu.cn (F.D.); hourz24@mails.jlu.edu.cn (R.H.); 2 School of Pharmaceutical Sciences, Jilin University, Changchun 130021, China; hanb@jlu.edu.cn

**Keywords:** bacterial extracellular vesicles, cell membrane-bionic nanoparticles, extracellular vesicles, gels, nasal–cerebral delivery

## Abstract

Central nervous system (CNS) diseases exhibit high incidence rates, and the blood–brain barrier (BBB) poses a major obstacle to drug delivery. Conventional drug delivery methods not only show limited therapeutic efficacy but also cause significant side effects. Intranasal administration offers a new strategy for CNS therapy by bypassing the BBB through the unique nasal-brain pathway, while nanodrug delivery systems (NDDSs) can improve drug delivery efficiency. On this basis, biomimetic drug delivery systems (BDDSs) based on cell membrane structure have been developed. The combination of nanoparticles modified by cell membranes or cell membrane-derived vesicles with carriers such as hydrogels creates a drug delivery system that utilizes a unique transnasal-to-brain pathway, opening new avenues for treating CNS disorders. This paper systematically reviews the classification, characteristics, and preparation strategies of BDDSs, while analyzing the anatomical pathways and physiological mechanisms of nasal–cerebral delivery. Furthermore, it delves into the biogenesis mechanisms of extracellular vesicles (EVs) and bacterial extracellular vesicles (BEVs). For CNS disorders, including glioblastoma multiforme (GBM), ischemic stroke (IS), Alzheimer’s disease (AD), and Parkinson’s disease (PD), this paper presents diverse applications and challenges of BDDSs in nasal–cerebral delivery.

## 1. Introduction

Disorders of the central nervous system (CNS) have been identified as one of the leading causes of death and disability worldwide [1]. It is estimated that there will be around 135 million people affected by CNS diseases toward the end of the century as reported by the Global Burden of Disease (GBD) study [2]. The mortality burden of these diseases is expected to surpass the burden caused by malignant neoplasms and to become the second killer after cardiovascular disease around 2040. The CNS can be affected by numerous disorders such as glioblastoma multiforme (GBM), ischemic stroke (IS), Alzheimer’s disease (AD), and Parkinson’s disease (PD). However, due to the diversity of types and complex causes, few effective clinical drugs have been developed for these diseases [3]. In particular, the existence of the blood–brain barrier (BBB) hinders the effective transport of drugs into the CNS [4]. The BBB is formed by brain microvascular endothelial cells, astrocytes, pericytes, and a basement membrane [5]. It only allows small molecules with molecular weights < 500 Da and high lipid solubility to pass through and actively expels large-molecule drugs and polar compounds [6]. As a result of the natural BBB, the drug concentration in the brain is extremely low when administered through traditional systemic routes such as oral and intravenous [7]. It leads to low therapeutic effectiveness and a large number of side effects [8]. Therefore, designing an efficient and safe CNS drug delivery system becomes of great importance and hot spot in the field of neuroscience [9].

In recent years, intranasal drug delivery has proven to be a non-invasive approach that enables drugs to bypass the BBB and enter the CNS effectively [10,11,12]. Drugs can enter the brain and cerebrospinal fluid (CSF) through the nasal epithelium or across the axons of olfactory and trigeminal nerves [13]. Compared with systemic administration and topical application, intranasal drug delivery is increasingly attracting attention because of its advantages, such as convenience, low systemic exposure, rapid onset of action, and high bioavailability [14].

With the rapid development of nanotechnology, nanodrug delivery systems (NDDSs) have become an important way to improve drug delivery efficiency and treat diseases more effectively [15]. The advantages of NDDSs are that they can greatly improve the solubility and bioavailability of hydrophobic drugs, greatly prolong the circulation time of drugs in the body, and improve the distribution of drugs therein [16]. Although NDDSs can target the lesion area through surface modification of antibodies and peptides [17], they are still limited in their application in clinical practice. Their poor permeability through the BBB and large degree of immunogenicity restrict the clinical application of NDDSs. To solve this problem, inspired by natural biological systems, a biomimetic drug delivery system (BDDS) based on cell membranes has been developed [18]. This technology combines the advantages of targeted modification and controlled release of NDDS and the characteristics of natural cell escape and active migration by encapsulating cell membranes on the surface of NDDS nanoparticles [19]. Combining a biomimetic cell membrane drug delivery system with a gel carrier fixed to the nasal cavity can prolong the duration of action, enabling sustained drug release and targeted delivery to the brain. It opens up a new route for the accurate and safe delivery of drugs to the CNS.

In this review, we systematically classify and organize the classification and characteristics, preparation, and application of BDDSs based on cell membranes, with further discussion on the biogenesis mechanism for two kinds of important membrane derived carriers: extracellular vesicles (EVs) and bacterial extracellular vesicles (BEVs). Meanwhile, the anatomical pathway and physiological mechanism of nasal–brain delivery are briefly described, particularly focusing on the wide range of applications of these BDDSs in nasal–cerebral delivery.

## 2. Cell Membrane-Based Bionic Nanodrugs

To pierce the BBB and combat the inflammatory damage commonly accompanying CNS diseases, BDDSs integrate the characteristics of multiple kinds of natural cell membranes and the characteristics of nanocarriers with great efficacy in drug delivery. One advantage of nanoparticles is their enhanced biocompatibility, immune evasion functions [20], and active target identification in the diseased area (because the natural antigens, receptors, and adhesion molecules remain on the surface of the cell membrane), whereas another advantage is that the nanoscale effect of the nanoparticles makes it easier to pass through the gaps in vascular endothelial cells or to use the natural antigens, receptors, and adhesion molecules on cell membranes to mediate endocytosis and pass through the BBB [21] and to control release and deliver drugs to target sites. Therefore, researchers have successively designed and prepared biomimetic nanodelivery systems based on modified materials such as red blood cell membranes, platelet membranes, macrophage membranes, neutrophil membranes, and stem cell membranes [22,23] to deliver drugs to brain lesions by taking advantage of the characteristics of diseases in the CNS [24].

### 2.1. Types of Cell Membranes

#### 2.1.1. Red Blood Cell Membranes

Red blood cells (RBCs) are intrinsic in blood, with a simple structure, low cost, and availability. They can stay in circulation for 120 days at most as the most numerous cells, which take part in the process of transporting O_2_ and CO_2_ [25]. The surface of the RBC membrane expresses glycoprotein CD47, and it can avoid phagocytosis of macrophages [26]. Thus, the circulation time of RBC carriers is prolonged due to their excellent biocompatibility [27] and long circulation period [28]. Liu et al. prepared a novel reagent by modifying the tumor neovascularization targeting peptide Arg-Gly-Asp (RGD) to red blood cells and then enriched an ICG-BSA nanocomposite (photothermal agent) into red blood cells through low perfusion dialysis [29]. In addition, Hu et al. invented a “top-down” biomimetic technique in 2011. They physically extruded degradable Poly (lactic-co-glycolic acid) (PLGA) nanoparticles into intact erythrocyte membranes [30]. Immune checkpoint proteins like CD47 were successfully anchored onto the surface of the nanoparticles, and their half-lives were noticeably longer than those of polyethylene glycol (PEG)-coated particles. This strategy was successful in creating a new long-circulation drug delivery system.

#### 2.1.2. Platelet Membranes

Platelets, the smallest blood cells formed from megakaryocytes [31], are widely known for their involvement in coagulation, but they are also an important component of the immune system, participating in both innate and adaptive immune responses [32]. Utilizing the intrinsic characteristics of platelet membranes, biomimetic drug delivery devices have exceptional natural targeting capabilities [33]. Membrane protein domains like CD62p and CD41 on the platelet surface help nanoparticles avoid immune clearance and allow for targeted delivery to specific locations, including tumors, blood clots, and inflammatory regions [24,34]. Platelet-coated nanoparticles are among the most promising targeted probes for the treatment of IS, as they are particularly well-suited for delivery to thrombi [35].

#### 2.1.3. Macrophage Membranes

As the first line of defense in the innate immune system, macrophages identify and destroy pathogens and necrotic or apoptotic cell debris in a physiological setting [36]. In addition to presenting antigens, they take part in immune surveillance and adaptive immunity. To preserve homeostasis, monocyte-derived macrophages polarize into M1 or M2 types when stimulated. There are many M1 macrophages present in early inflammation and tumors. These pro-inflammatory cytokines released by M1 macrophages help to defend against pathogens and resolve inflammation as well as induce anti-inflammation [37]. M2 macrophages release anti-inflammatory substances to promote inflammation resolution, tissue remodeling, and wound healing. Such an inflammation-regulated feature makes it a perfect biomaterial for treating neuroinflammatory diseases [38]. As the cell membrane retains its inherent immune recognition properties (e.g., adhesion to damaged blood vessels/inflammatory environment), it can be further modified on the surface of cell membranes for targeting or drug loading through surface modification technology [39]. This provides a new solution to the shortcomings of traditional nanodelivery systema in BBB penetration, target localization, and modulation of the immune microenvironment [40].

#### 2.1.4. Neutrophil Membranes

Neutrophils are the most abundant cells in human peripheral blood, comprising 40% to 60% of the total population of white blood cells in healthy individuals. As a crucial component of innate immunity, neutrophils are among the first cells to combat pathogens [41]. Due to this characteristic, the nanoparticles derived from neutrophil membranes retain their innate immune recognition properties, exhibit superior biocompatibility, higher drug loading amounts, and stronger targeting properties, and avoid the problems brought by live cells, such as high immunogenicity and short survival period [42].

In the inflammatory environment, there also exist many diffusible mediators released by inflammatory cells, such as granulocyte colony-stimulating factor (G-CSF), transforming growth factor-β (TGF-β), interleukin-6 (IL-6), and intercellular adhesion molecule-1 (ICAM-1). These mediators can cause the neutrophil membrane-coated nanoparticles to migrate to the inflammatory environment and suppress the inflammatory cascade reaction at the diseased site [43]. At present, the neutrophil membrane can effectively load liposomes, polymers, and inorganic nanomaterials to provide new choices for the treatment of diseases [44,45,46].

#### 2.1.5. Stem Cell Membranes

As a new biomaterial, stem cell membrane-modified nanoparticles show clear benefits in BBB penetration, targeting ability, and immune evasion [47]. First of all, stem cell membranes preserve the special “self-recognition” properties of stem cells while acting as organic encapsulation materials for nanoparticles [48]. This makes it possible for autologous cells to be effectively mimicked after they reach the body, greatly lowering the likelihood of immune system identification and attack [49]. Long-term treatment safety is thus ensured by minimizing immunological rejection. Second, to achieve precise tumor homing, stem cell membrane-modified nanoparticles use chemokine receptors (like CXCR4) that are widely distributed on the membrane surface of stem cells to react to chemokines (like SDF-1) that are highly expressed in sick locations [50,51]. This improves treatment efficacy and lowers systemic toxicity by allowing high-concentration drug accumulation in target locations. Furthermore, stem cell membrane-modified nanoparticles significantly increase the effectiveness of nanodrug penetration across the BBB by interacting with BBB endothelial cells through chemokine receptors and stem cell membrane adhesion molecules [52]. This opens up new treatment options for conditions affecting the CNS. Table 1 summarizes the aforementioned cell membrane types, their respective advantages and drawbacks, and their applications in CNS diseases.

### 2.2. Preparation of Cell Membrane-Modified Nanocarriers

#### 2.2.1. Cell Membrane Extraction and Purification

One of the most important steps in creating cell membrane-modified nanocarriers is the extraction and purification of cell membranes [20]; the techniques used must be tailored to the specific cell type. Lysis and purification are usually the two main steps in the fundamental procedure of cell membrane extraction [24]. One of the most commonly used lysis methods is hypotonic treatment, after which sucrose gradient centrifugation, differential centrifugation, and repeated freeze–thawing are used to obtain cytoplasmic and nuclear fractions [57]. The final membrane fraction is then obtained with specific buffers or eluents from the sample.

It is relatively easy to obtain membranes from anucleate cells [58]. For instance, anucleate cells like RBCs and platelets can be lysed with hypotonic solutions or repeated freeze–thawing, and then cell membranes are collected via centrifugation [59]. However, for eukaryotic cells, especially small cells with specially structured surface proteins, the combination of hypotonic treatment and repeated freeze–thawing and mechanical methods (homogenizers, ultrasonication, etc.) are used to lyse eukaryotic cells [60]. Lastly, the final membrane structure is obtained by removing intracellular components via differential centrifugation or discontinuous sucrose density gradient centrifugation, followed by sterile filtration [61,62]. For example, the most commonly used methods to obtain neutrophil membranes are density gradient centrifugation and flow cytometry sorting [63]. Density gradient centrifugation uses media with different densities to remove cells from bone marrow, tissues, peritoneal fluid, and peripheral blood. Flow cytometry sorting uses fluorescently labeled specific antibodies to detect specific proteins on the cell membrane and sort cells via flow cytometry to obtain higher purity isolates of neutrophils. After cell extraction, membrane isolation necessitates cell lysis, which is often accomplished by means of cryogenic disruption, high-pressure homogenization, or ultrasonication. Lastly, the cell membranes are purified via differential centrifugation.

#### 2.2.2. Membrane–Nanocarrier Fusion

**Figure 1:**
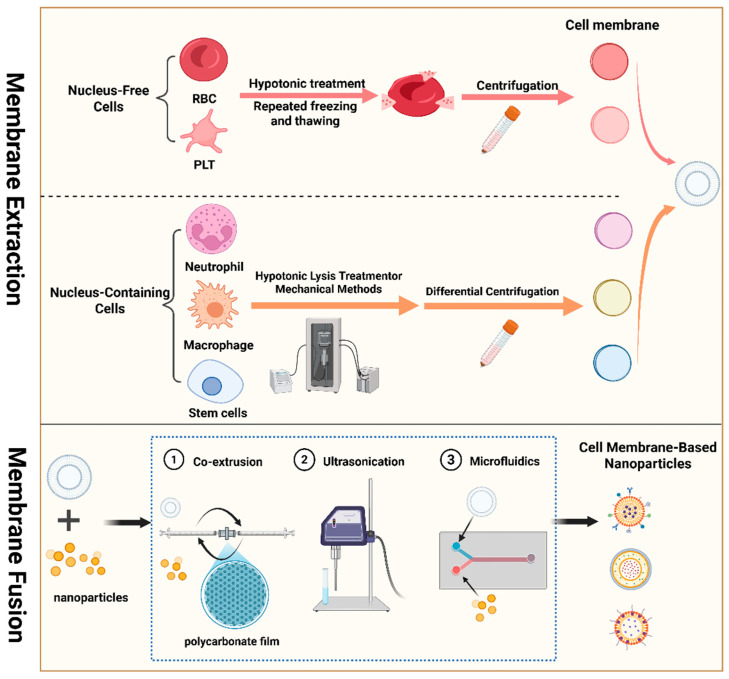
Processes of cell membrane extraction and membrane–nanocarrier fusion for constructing cell membrane-modified nanocarriers. For anucleate cells, cell membranes are obtained through hypotonic treatment, repeated freezing–thawing, and centrifugation. For nucleated cells, cell membranes are extracted by means of hypotonic lysis, mechanical methods (such as homogenization), and differential centrifugation to remove intracellular components. The extracted cell membranes are fused with nanoparticles via three core methods: co-extrusion, ultrasonication, and microfluidics. (Created in BioRender, Ding, F (2025) https://BioRender.com/17gkzfg).

Membrane-mimetic nanomedicines are created by fusing extracted cell membranes with nanocarriers, as shown in Figure 1. In the field of fusion technologies between nanocarriers and cell membranes, co-extrusion, ultrasonication, and microfluidic electroporation stand as three widely applied core methods [64]. They achieve fusion based on distinct mechanisms of action, each with unique focuses in terms of operational logic and performance.

The co-extrusion technique leverages the mechanical force of the extrusion process to directly embed and encapsulate nanocores within the cell membrane structure [65]. Cell membranes and nanoparticles are suspended in the same dilution buffer and physically squeezed back and forth through a polycarbonate membrane to form stable and uniform cell membrane-derived biomimetic carriers. However, low yield and cumbersome preparation procedures limit the application of this method. Ultrasonication is a commonly used method in laboratories. Ultrasonic treatment applies forces that disrupt cell membranes, allowing membrane fragments to randomly coat nanoparticles or be incorporated into phospholipids [66]. This method is simple, requires minimal equipment, and involves lower material costs. Currently, particularly in cell membrane-coated nanotechnology, ultrasonication is more suitable for nanoparticles with high mechanical strength, such as PLGA nanoparticles or metal nanoparticles. However, during the ultrasonic treatment process, functional proteins in cell membranes may denature due to excessive temperatures, severely impairing their biological properties [60].

Microfluidic electroporation technology relies on an applied electric field to break down the dielectric layer on the cell membrane, creating multiple transient pores that allow biomolecules and nanoparticles to enter the cell membrane, thereby achieving the preparation of cell membrane-coated nanoparticles [52]. According to the properties of vesicles and nanoparticles, the channel, voltage, and flow rate are chosen. Subsequently, vesicles and nanoparticles are injected into the microfluidic chip separately [67]. Finally, the mixture collected from the outlet of the microfluidic chip is the cell membrane coated nanoparticles after the electroporation. This approach can enable current control and high throughput and the preparation of cell membrane-coated nanoparticles in one step with high coating efficiency while maintaining the function of the membrane protein. However, microfluidic technology faces limitations in various laboratories. Due to ongoing technical constraints in microfluidic chip design and high equipment requirements, its widespread adoption remains challenging [68].

In summary, appropriate preparation methods should be selected according to the properties of drugs and therapeutic requirements to prepare uniform cell membrane-coated nanomedicines.

## 3. Bionic Nanomedicines Based on Cell Membrane-Derived Vesicles

### 3.1. Extracellular Vesicles (EVs)

Extracellular vesicles (EVs) are naturally occurring nanovesicles carrying diverse bioactive molecules, valued for their excellent biocompatibility, low immunogenicity, and superior physicochemical properties [69]. EVs can mediate signal transduction by transferring bioactive molecules to cells in target organs or tissues through the bloodstream [70,71]. EVs originate from diverse sources, with human body fluids and tissues extensively studied, including blood, urine, saliva, cerebrospinal fluid, tears, and feces [72]. Among these, blood-derived EVs are currently the primary focus of research. Blood EVs, carrying molecular information from their source cells, have become a key subject in liquid biopsy studies [73]. Another significant source is the culture supernatant of mammalian cells, such as immune cells and dendritic cells [74]. However, during cultivation, these cells secrete not only EVs but also other metabolites, presenting drawbacks of low yield and high heterogeneity [75]. Furthermore, the academic community is increasingly focusing on EVs derived from milk, plants, and bacteria [76]. These sources are highly suitable for large-scale production due to their low cost and high yield characteristics. The following paragraphs mainly introduce EVs from different cellular sources in the blood, including their characteristics and preparation methods.

#### 3.1.1. Red Blood Cell-Derived Extracellular Vesicles

Because RBCs are the most populous cell population in blood, it is easy to obtain red blood cell-derived extracellular vesicles (RBCEVs) and produce a large amount of them [77]. They are low immunogenicity, highly biocompatible, and harmless by nature. Since mature RBCs do not contain nuclear and mitochondrial DNA, RBC-EVs are less likely to carry bioactive chemicals, which reduce the risk of oncogenic phenotypes or horizontal gene transfer and enhance safety [78]. RBCEVs express surface molecule CD47, which could interact with macrophage inhibitory receptor SIRPα to reduce the negative inflammatory reaction, block phagocytosis, and reduce the clearance in circulation [79]. RBCEVs are generally nanoscale in size and contain high levels of lipids, proteins, and miRNAs. Biomolecules, nucleic acids, and small-molecule medications are among the therapeutic compounds that can be effectively enclosed and protected by RBCEVs. For instance, this study demonstrated the potential of the RBCEV platform as a viable and safe method for delivering nucleic acids to the CNS. They observed that RBCEVs administered via intrathecal injection exhibited extensive distribution throughout the CNS and were efficiently taken up by neuronal cells. Delivery of RBCEVs carrying a green fluorescent protein (GFP)-encoding plasmid resulted in GFP expression within neurons [80].

Another obvious advantage of the inherent capacity of RBCEVs to cross the BBB and enter the brain without surface modification is that transferrin receptor (TfR) and transferrin–transferrin receptor interactions are involved in this process and are accelerated in inflammatory milieu [81]. In acute or chronic neuroinflammatory diseases in which the BBB may be weakened or the inflammatory milieu is actively promoting EV uptake, this finding suggests that RBCEV treatments may be particularly useful [82]. This information will help select the appropriate patient or decide when to treat a patient for a better outcome in stroke, traumatic brain injury (TBI), or other neurodegenerative diseases with high neuroinflammatory activity. RBCEVs have broad anti-inflammatory effects [83] and are thus ideal neuroinflammatory modulators for the CNS. They show potential in reducing neuronal cell death and in neurodegenerative diseases (such as AD and PD) and preserving neuronal function due to their ability to naturally stimulate anti-inflammatory responses in macrophages and regulate the release of pro-inflammatory cytokines (IL-6 and IL-1β).

#### 3.1.2. Platelet-Derived Extracellular Vesicles

The most common type of EVs in blood are platelet-derived EVs (pEVs), which can be found in large amounts in patients with TBI [84]. They have platelet-like or even more extensive functions because they are byproducts of platelets. Lipids, proteins (such as IL-1β, IL-6, IL-8, and tumor necrosis factor [TNF]), microRNAs, and organelles like mitochondria are among the several substances carried by pEVs [85]. They are involved in several biological processes, such as inflammation and coagulation malfunction, which are crucial for subsequent damage after traumatic brain injury. P-selectin (CD62P) and common activation markers such as platelet-derived markers CD41 and CD63 can be used to identify EVs generated by activated platelets [86]. In addition, research indicates that EVs present in human platelet lysate derived from clinical-grade platelet concentrates exhibit distinct neurogenic capabilities and may serve as an independent therapeutic modality for regenerative neurology [87].

#### 3.1.3. Macrophage-Derived Extracellular Vesicles

As mentioned before, there are two different phenotypes of macrophages: pro-inflammatory M1 and anti-inflammatory M2 [88]. M2-derived EVs reduce secondary injury, boost neurological healing, improve phagocytosis, assist axonal myelin regeneration and elongation, and keep the blood-spinal cord barrier intact [89]. Levels of anti-inflammatory cytokines (TGF-β, IL-4, and IL-10) are increased whereas those of pro-inflammatory cytokines (TNF-α, IL-1β, and IL-6) are decreased. Macrophage EVs have a lot of promise for precision medicine in treating CNS illnesses, according to engineered M2-EVs [90]. Beyond the constraints of broad-spectrum anti-inflammatory medications, therapies can highly focus inflammation reduction, tissue repair promotion, and neuroprotection enhancement by selectively encouraging M2-EVs or designing them to carry specific cargo (e.g., GDNF and melatonin pre-treatment).

#### 3.1.4. Neutrophil-Derived Extracellular Vesicles

Two different EV subtypes are produced by neutrophils: neutrophil-derived microvesicles (NDMVs) and neutrophil-derived “trajectories” (NDTRs) [91]. Neutrophils that are already at the site of inflammation make NDMVs, while neutrophils migrating towards inflammatory foci produce NDTRs. The integrin signaling pathway is responsible for the formation of NDTRs, which are regarded as pro-inflammatory EVs because they carry pro-inflammatory miRNAs that can cause M1 macrophage polarization [92]. On the other hand, the PI3K pathway is necessary for the production of NDMVs, which are anti-inflammatory EVs that cause M2 macrophage polarization.

#### 3.1.5. Stem Cell-Derived Extracellular Vesicles (SC-EVs)

EVs produced from stem cells, such as mesenchymal stem cell-derived extracellular vesicles (MSC-EVs), stimulate angiogenesis and neurogenesis in a variety of CNS injury models, including stroke, spinal cord injury, and AD [93,94]. They promote synaptic development, neurite remodeling, and the proliferation and differentiation of neural stem cells. The strong anti-apoptotic and neuroprotective properties of MSC-EVs and NSC-EVs protect neurons from damage and cell death. Normally, this involves stimulating autophagy and modulating signaling pathways (such as PI3K/AKT, Wnt/β-catenin, and JNK/c-Jun) [95]. Another notable advantage of SC-EVs resides in their potent anti-inflammatory and immunomodulatory effects [96]. MSC-EVs can promote the differentiation of regulatory T cells (Treg), inhibit the activity of Th17 cells, and regulate the signaling pathways of dendritic cells (DCs). Genetically engineered MSC-EVs overexpressing miR-540-3p can suppress the expression of the CD74/NF-κB axis, decrease the expression of inflammatory mediators IL-1β and IFN-γ, and simultaneously enhance the expression of anti-inflammatory factors IL-10 and TGF-β1, thus alleviating inflammatory damage. Because SC-EVs as nanoscale microvesicles can pass through the BBB easily, using SC-EVs to repair and regenerate intracranial or intraspinal CNS lesions is another pioneering research direction for the treatment of CNS injuries. For example, MSC-EVs modulate the immune response of amyloid-β (Aβ)-activated microglia, which in turn ameliorate the microenvironment of neuronal survival in the brain tissue of AD patients [97]. Research on the brains of AD transgenic mice shows that MSC-EVs suppress the polarization of glial cells toward the pro-inflammatory M1 subtype and increase the proportion of anti-inflammatory M2 subtype glial cells. At the same time, MSC-EVs upregulate the expression of anti-inflammatory factors TGF-β and IL-10 in the brain tissue of AD transgenic mice [98]. And this immunomodulation may also be involved in the neuroprotective effect of MSC-EVs on AD neurons.

#### 3.1.6. Biogenesis of Extracellular Vesicles

According to their intracellular origin, EVs can be mainly divided into three categories: exosomes, microvesicles, and apoptotic bodies [99]. Exosomes are vesicles with a diameter of approximately 30–150 nanometers. When the membrane of the endosome buds inward to form an exosome, the endosome generates multivesicular bodies (MVBs). When MVB fuses with the plasma membrane, exosomes are released into the extracellular space. Microvesicles (MVs) range in size from 100–1000 nm in diameter and are produced by direct budding and shedding from the plasma membrane [100]. This process involves local changes in the composition of plasma membrane proteins and lipids, which induce the invagination of the plasma membrane. Apoptotic bodies are the largest, ranging from 500–4000 nm in diameter and often appear during apoptosis, containing intracellular material and organelles [101].

#### 3.1.7. Preparation and Separation Methods for Extracellular Vesicles

It is important to separate EVs from the physiological fluid or supernatant of cell culture medium to study and use them. The common methods of separation are as follows [102]: (1) Differential Ultracentrifugation: It is also known as the standard gold method for separating EVs [103]. This method gradually removes impurities such as dead cells, cell debris, platelets, protein complexes, and nucleic acids by centrifuging a certain number of times at different speeds to obtain relatively pure EVs. (2) Density Gradient Ultracentrifugation (DGUC): Separation according to density differences, often used in combination with differential centrifugation. This method enables the separation of low-density EVs from other vesicles, granules, and contaminants, yielding EVs of high purity [104]. (3) Polymer Precipitation: This method uses hydrophilic polymers (such as PEG) to make EVs less soluble, which causes them to precipitate and facilitates separation. This method yields more EVs, but there may be protein contaminants. (4) Ultrafiltration: Use membranes with different diameters of pores to screen EVs. EVs are larger than typical proteins. By employing ultrafiltration membranes with different molecular weight cut-offs, samples can be selectively separated. Soluble proteins and particles smaller than the critical threshold (approximately 105 kDa) are directed toward the membrane, where EVs are subsequently collected [102]. (5) Immunoaffinity Isolation: Highly specific separation is made possible by antibody-conjugated magnetic beads that selectively capture specific EV surface markers. This method is simple to perform, highly specific, does not affect the integrity of EVs morphology, yields acceptable purity, and allows the obtained EVs to be used directly for analysis or for DNA or total RNA isolation [105]. (6) Size Exclusion Chromatography (SEC): High-purity samples are produced via separation using chromatography columns based on EV size, but sample dilution may occur. (7) Microfluidics Techniques: Microfluidic technology employs nano-scale channel chips to separate EVs using physical fields such as fluid dynamics, surface affinity, electric fields, or acoustic fields [106]. Rapid, low-sample-volume EV separation is made possible by the use of microfluidic chips.

Since EVs from various cellular origins have distinct compositions and roles, there may be variations in the emphasis placed on their preparation techniques. During erythropoiesis, cellular aging, or under particular activation conditions, red blood cell EVs are spontaneously created. To generate them in vitro, erythrocytes can be treated with phorbol esters and then differentially centrifuged [107]; in other investigations, erythrocytes are budded to form EVs using Ca^2+^-EDTA [108]. Activated platelets are the primary source of platelet-derived EVs (pEVs), with platelet activation during the manufacture and storage of platelet concentrates serving as the primary catalyst for pEV synthesis [109].

### 3.2. Bacterial Extracellular Vesicles

A bidirectional information exchange network between the gut microbiome and the brain is becoming more and more evident in clinical data as studies into the body’s intrinsic pathways advance [110]. The gut microbiota plays a critical role in the development and function of the CNS [111]. It has been shown in the past few years that EVs secreted by living cells are highly efficient drug carrier vehicles. Now it is becoming increasingly clear that probiotics modulate the functions of distal organs by releasing EVs that mediate host signaling pathways’ modulation and carry bioactive compounds to target host cells [112]. Among the many benefits of bacterial EVs (BEVs) are their tiny width, lack of cellular structure, excellent biocompatibility, and adaptability. Bacteria have several benefits, including advanced high-density culture techniques, varied gene editing approaches, and fast multiplication [113]. Therefore, using BEVs as medicine delivery vehicles provides a way around the difficulties in producing EVs derived from mammals on a big scale.

#### 3.2.1. Origin of Bacterial Extracellular Vesicles

BEVs are spherical, bilayer membrane structures that range in diameter from 20 to 400 nm and are actively released by bacteria [114]. Their lumens serve as a “Trojan horse”-like secretory system for bacteria, concentrating parent bacterial outer membrane proteins, periplasmic proteins, peptidoglycan, lipopolysaccharides, phospholipids, DNA, RNA, and different virulence factors [115]. Initially misunderstood as “cellular debris” following bacterial lysis, these vesicles were first seen by means of electron microscopy in 1965 in Escherichia coli and later verified in Vibrio cholerae [116]. It was long believed that thick-walled bacteria could not produce BEVs because of their thick peptidoglycan walls, which are a feature of Gram-positive bacteria. In 2009, this misconception was disproved when vesicles from Gram-positive bacteria, including Staphylococcus aureus, were successfully isolated. Similar structures were later found in archaea, indicating that BEVs are a conserved secretory system that connects the bacterial and archaeal domains [117]. Nowadays, BEVs not only participate in the environmental adaptation and evolution of bacteria themselves but are also closely associated with human physiological homeostasis and the onset and progression of various diseases.

#### 3.2.2. Mechanisms of Bacterial Extracellular Vesicle Formation

**Figure 2:**
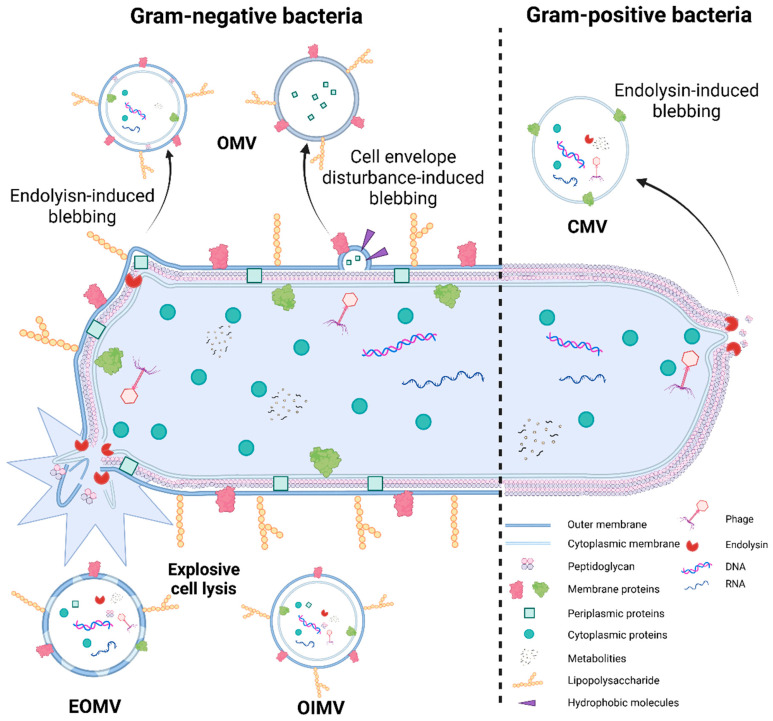
Mechanisms of bacterial extracellular vesicle (EV) formation in Gram-negative and Gram-positive bacteria. Gram-negative bacteria generate OIMVs and EOMVs via cell lysis and OMVs via outer membrane vesiculation. Gram-positive bacteria produce CMVs through endolysin-induced peptidoglycan breakdown, with vesicles containing cytoplasmic and membrane components (Created in BioRender, Ding, F (2025) https://BioRender.com/x9xa27x).

BEVs have a highly type-specific biogenesis, with different production processes depending on the architecture of the bacterial cell wall. Figure 2 presents hypotheses about the mechanisms of formation of different forms of EV.

Gram-negative bacteria primarily generate EVs via two core pathways: firstly, through outer membrane budding to form outer membrane vesicles (OMVs); secondly, through cell lysis to generate outer-inner membrane vesicles (OIMVs) and explosive outer membrane vesicles (EOMVs) [118]. Either an imbalance in peptidoglycan production or the entrance of hydrophobic molecules into the outer membrane—both of which cause membrane disruption—are the triggers for outer membrane budding [119]. These situations result in localized outer membrane protrusions that separate from the parent cell and eventually generate OMVs. As OMVs originate from the outer membrane, their composition is rich in outer membrane proteins and exhibits specific lipid composition characteristics [120].

In Gram-negative bacteria, there are two ways that explosive cell lysis starts. The first is when the peptidoglycan layer is broken down by autolysin, which causes the inner membrane to emerge [119]. DNA and other cytoplasmic materials then enter the vesicular structure, which separates from the cell surface along with the outer membrane to create OIMVs. The second pathway is when oxidative stress brought on by bacterial chromosomal DNA damage causes cell death lysis. Here, pieces of cell membranes aggregate and recycle, encasing cytoplasmic material at random to create EOMVs. Cell membrane vesicles (CMVs) are vesicles produced by Gram-positive bacteria as opposed to Gram-negative bacteria [121]. Their primary mechanism of production is the defective-prophage-encoded endolysin, which breaks down the bacterial peptidoglycan layer and causes the cell membrane to enclose the cytoplasmic contents and protrude outward, resulting in the formation of CMVs [122]. Both cytoplasmic and cell membrane components are present in these vesicles. To more systematically compare the characteristics of membrane and membrane vesicle carriers from different sources, and clarify their differences in relevant research and applications, Table 2 elaborates on them in detail from aspects including nature, source, preparation, and main advantages.

#### 3.2.3. Preparation, Advantages, and Disadvantages of Bacterial Extracellular Vesicles (BEVs)

The preparation of BEVs boasts significant advantages in large-scale production, with bacterial culture and purification as the core processes [124]. Bacteria are first cultured through methods such as fermentation; once a sufficient quantity of bacteria is obtained, BEVs are isolated and extracted via conventional purification techniques including centrifugation, filtration, and density gradient centrifugation [123]. Owing to the rapid reproduction rate of bacteria and their simple culture requirements (no complex nutrient systems are needed); the entire process enables convenient large-scale production.

BEVs also exhibit immunomodulatory effects. Toll-like receptors (TLRs) are one type of PRR located on the surface of immune cells [125]. On the surface of BEVs are pathogen-associated molecular patterns (PAMPs), such as lipopolysaccharide (LPS), viral RNA, and bacterial DNA; these PAMPs can be recognized by TLRs on immune cells. These signals mediate the immune response that protects the body from pathogen infection. Meanwhile, Damage-Associated Molecular Patterns (DAMPs) contained in BEVs can also be recognized by TLRs. Since BEVs can stimulate both the innate and adaptive immune responses, they can be used as vaccine candidates [126]. One BEV can encapsulate and load multiple antigens and present them in their native conformation, which is beneficial for immunogenicity. In addition, due to the nanoscale size and biocompatibility of BEVs, they can be used as targeted delivery systems for vaccines to load vaccine components to specific immune cells and improve uptake and immunogenicity.

Application of bacterial EVs also faces problems related to toxicity. Since the PAMPs (especially LPS) on the surface of bacterial EVs can induce immunostimulation effects, if the degree of activation is too high or the regulation is inappropriate, the immune cells will be activated and induce toxic reactions in the body [122]. These toxic reactions can threaten the biological safety of the body. Therefore, before the practical application of bacterial EVs, we should take certain treatment strategies to reduce the toxicity of bacterial EVs.

## 4. Applications of Bionic Nanodrugs Based on Cell Membranes and Vesicles in CNS Diseases

Bionic nanodrugs based on cell membranes and vesicles have shown remarkable potential in addressing the key challenges of CNS diseases, such as poor BBB penetration, insufficient targeting ability, and limited therapeutic efficacy. Different types of bionic materials exhibit distinct core functions and specific mechanisms in the treatment of GBM, IS, AD, and PD. An overview of their applications in various neurological diseases is summarized in Table 3.

### 4.1. Glioblastoma Multiforme

Glioblastoma multiforme (GBM), the most common primary malignant tumor of the CNS, poses three main therapeutic challenges: a complex tumor microenvironment, medication resistance, and the BBB [140]. First, the BBB hinders drugs from entering the CNS; second, using only one chemotherapeutic agent alone results in resistance in a short time, and this prompts us to combine it with new tumor killing methods [141]; in addition, the tumor microenvironment of GBM promotes its rapid growth and tissue infiltration quickly, so we think that it is indispensable in GBM treatment [142]. A large number of clinical issues remain to drive gliomas toward more effective diagnostic and therapeutic synergy with the precise modulation of tumor-specific molecular pathways for further medication development, which is still in progress.

Liu et al. synthesized Ang-RBCm-CA/siRNA nanoparticles. RBCm, a biomimetic carrier, can protect the siRNA [127]. After Angiopep-2 modification, the nanoparticles can improve BBB penetration and GBM targeting. These particles show long-circulation and tumor-targeting properties, and the siRNA can be burst released in the acidic microenvironment of the tumor, which can realize long-term drug delivery with less dosage and improved therapeutic efficacy, providing a new strategy for glioma therapy. Wang et al. constructed engineered macrophage membrane coated nanocarriers carrying programmed cell death protein-1 (PD-1) for glioma treatment [128]. The in vitro BBB simulation and in vivo real-time bioluminescence fluorescence imaging results showed that the PD-1-MM@PLGA/RAPA biomimetic nanoparticles could achieve effective BBB penetration and tumor accumulation.

Based on the characteristics of the pathogenesis of brain tumors, a research team propose using extracellular vesicles (M1EVs) derived from M1 macrophages as a carrier [129]. The phenotype switching of macrophages can control the immune response of the tumor microenvironment, and taking advantage of the chemotactic characteristics of M1 macrophages, these vesicles will be widely concentrated at GBM sites. Moreover, they loaded chemically responsive molecular pairs (CPPO and Ce6) and hypoxic drugs (AQ4N) into the cell membrane and lumen of M1EVs. Thus, tumor microenvironment regulation, chemoresponsive kinetics, and hypoxic tumor therapy were integrated into this delivery system.

Due to the advantages of using bacterial outer membrane vesicles (OMVs) as transporters, immunological adjuvants, and BBB crossing agents, they have many benefits for GBM treatment. Recently, researchers developed Fe_3_O_4_-MnO_2_ (FMO) nanoplatforms functionalized with bacterial OMVs to accomplish photothermally improved cancer immunotherapy and drug-free neutrophil targeted delivery [130]. Modified Escherichia coli OMVs are used in this particular strategy to promote the accumulation of FMO NP within tumors. The breakdown of FMO releases iron and manganese ions, which induces ICD and improves tumor hypoxia. Meanwhile, OMVs overcome immunosuppression and activate immunity through pathogen-associated molecular patterns. To support nanoparticle therapy, the photothermal impact immediately destroys tumors and increases inflammation.

### 4.2. Ischemic Stroke

A major contributor to persistent disability and the leading cause of death from cardiovascular disease [143], ischemic stroke (also called cerebral infarction) can account for up to 80% of all stroke occurrences [144]. Its main cause is cerebral atherosclerosis, a disease that reduces the flexibility and compliance of the blood vessels. The cerebral tissue blood supply is disrupted when plaques and thrombi block blood arteries, which results in a sharp drop in nutrients like oxygen and glucose and damages brain tissue [145]. Nowadays, thrombolytic drugs like t-PA are frequently used in clinical settings to restore blood flow to the brain. Nevertheless, t-PA has drawbacks, such as a limited therapeutic window (≤4.5 h) and a significant chance of side effects such as cerebral hemorrhage and reperfusion damage [146]. Traditional pharmaceutical treatments include lipid-lowering medications, neuroprotective measures, and antiplatelet medicines. However, these medications usually face BBB hurdles within the limited therapeutic window, which hinders their maximum effectiveness and causes significant disability or death for many patients [147]. Therefore, there is a pressing need for better therapeutic strategies to improve the diagnosis and treatment of stroke.

Using red blood cell membranes as the encapsulating material, the Lv team designed a polymeric nanoparticle system that is specifically designed to deliver the neuroprotective drug NR2B9C to stroke lesions, enabling intervention against ischemic brain injury [131]. The shell of this nanocarrier composed of erythrocyte membrane embedded with a homing peptide (SHp) for stroke site targeting and the core composed of a boronic ester modified dextran polymer with ROS responsive properties. Based on this design, the resulted SHp-RBC-NP nanoparticles can precisely localise to ischemic brain tissue and modulate the release of neuroprotective agent NR2B9C according to the microenvironment of the stroke lesion site.

Coating other therapeutic materials with platelet membranes is an important strategy for IS treatment. To solve the problems induced by platelet-activation resulting in thrombogenesis in IS as well as the bleeding and narrow therapeutic window caused by tissue Plasminogen Activator (tPA) treatment, researchers designed a tPA delivery platform (APLT-PA) based on Annexin V and platelet membrane. Prepared by extruding the platelet membrane and inserting membranin V into the lipid matrix, it shows high targeting specific towards activated platelets in vitro and thrombus sites in vivo via binding to phosphatidylserine (PS) and activated platelet membrane proteins [132]. In a photochemically induced acute IS mouse model, only one dose of APLT-PA made it possible to reach a significant thrombolytic effect for more than 7 days and greatly improved neurological function. In addition, researchers have developed platelet-derived nanocarriers to encapsulate and protect recombinant tissue Plasminogen Activator (rtPA) from degradation [148]. These carriers, composed of human platelet membrane fragments, present a particle size of 200 nm when loaded with rtPA. After lyophilization, the preparation in which only one bolus injection of the formulation was administered in a mouse model of stroke thromboembolism showed similar efficacy to the continuous clinical infusion of free rtPA at an equivalent concentration; meanwhile, it did not increase the risk of hemorrhagic transformation and did not cause an inflammatory response, showing favorable biocompatibility.

To counter the oxidative stress caused by ischemia–reperfusion, scientists have designed a multi-level targeted delivery system—SNM-NPs. This system, which involves coating nanoparticles within neutrophil membranes and appending a stroke homing peptide (e.g., the RVG peptide, which localizes to the ischemic penumbra)—bestows the nanoparticles with dual targeting abilities for oxidative stress responses and inflammatory chemotaxis [133]. The stroke homing peptide enhances the selectivity of the ischemic core, and the chemotactic characteristics of the neutrophil membrane guide the system to the injured area. Under the ROS milieu, the edaravone loaded inside is gradually released and cleverly scavenges free radicals, attenuates neuroinflammation, and inhibits neuronal death simultaneously. The precision-targeted therapeutic effects of SNM-NPs were further verified by in vivo investigations, which demonstrated a significant decrease in the infarct size in stroke models without any observable side effects of systemic toxicity.

While post-stroke brain regeneration is a long-term process, a single dose of free EVs is insufficient to provide long-lasting therapeutic benefits. Researchers creatively used hydrogel microspheres (MS) made using microfluidic technology as a reservoir for the sustained release of modified extracellular vesicles (S-EVs@MS) in order to overcome this difficulty [149]. After an IS, the goal of targeted intracerebral injection of these microspheres is to achieve long-term brain healing. For S-EV loading, they created MS with a diameter of about 200 μm, with an efficiency of up to 80%. According to in vitro release results, S-EVs were gradually and continuously released from the microspheres; after 15 days, 46% of them had been released. Furthermore, the microspheres’ outstanding biocompatibility was shown by in vitro cytotoxicity tests. The improved neuroprotective effects of S-EVs@MS were then confirmed by the researchers in a stroke animal model.

Zhou et al. prepared a new kind of medicinal carrier by employing outer membrane vesicles (OMVs) harvested from bacteria [134]. The method employed OMVs’ own LPS components to connect onto TLR4 receptors on the surface of neutrophils by loading pioglitazone (PGZ) onto the OMV surface to prepare OMV@PGZ. The challenge of drug penetration into the brain in IS was solved by effective transmigration of PGZ into the brain after uptake by neutrophils. The experimental results showed that OMV@PGZ simultaneously inhibited the activation of the NLRP3 inflammasome and the ferroptosis signaling pathway and alleviated the neuropathic damage caused by reperfusion and remarkably improved the therapeutic effect. In addition, Lactobacillus plantarum-derived extracellular vesicles (LEVs) have advantages of low cost and high yield. Another study revealed that LEVs can upregulate miR-101a-3p, which targets c-Fos and regulates ischemic neuronal apoptosis via the TGF-β1 pathway, thereby enhancing neural repair following stroke [135].

### 4.3. Alzheimer’s Disease

Alzheimer’s disease (AD) is one of the most common degenerative disorders of the CNS [150] characterized by gradual decline in cognitive functions often associated with psychiatric and behavioral symptoms, social functioning, and learning ability [151]. The pathophysiology of AD is largely due to the abnormal hyperphosphorylation of tau protein that results in its aggregation and segregation from the cytoskeleton and subsequently intracellular neurofibrillary tangle formation [152]. Moreover, AD pathogenesis is significantly influenced by reactive oxygen species (ROS)-induced neuronal mitochondrial dysfunction, inflammatory mediators, and neurotoxic chemicals generated by hyperactivated microglia [153]. Despite the development of multiple therapeutic strategies, AD remains an incurable, chronic, debilitating disease. The effectiveness of currently available clinical medications is still restricted in advanced stages. According to recent studies, one of the main factors limiting therapeutic success is ineffective medication distribution.

To tackle the problem of neurotoxic accumulation (such as β-amyloid) causing glial cell hyperactivation and neuroinflammation, a research team created an engineered macrophage-mimetic multifunctional nanodetoxifier (OT-Lipo@M); it targets inflammatory foci in the AD brain and actively crosses the BBB thanks to its encapsulated macrophage membrane’s pro-inflammatory and anti-phagocytic properties. The reduction in neurotoxic levels prevents aberrant activation of microglia. At the same time, oxytocin (OT), which is given gradually, inhibits TLR4-mediated pro-inflammatory signaling cascades by downregulating TLR4 expression in microglia. This approach offers new therapeutic insights for glia-mediated neuroinflammation and shows notable effectiveness in reducing cognitive impairments, preventing neuronal death, and postponing brain atrophy in AD mice [136]. Researchers recently combined metal–organic framework (MOF) nanoenzymes with neutrophil membranes to create a Neu-MOF/Fla nanoparticle. The nanoparticles can actively penetrate the BBB and target AD lesions because the chemokine receptors on the surface of neutrophil membranes (like CXCR2) bind to chemokines in the inflammatory milieu (like IL-8) [137]. In the meantime, the MOF-based nanozyme spontaneously scavenges reactive oxygen species (ROS) through its hydrolytic capabilities and produces the anti-inflammatory agent CO when light is triggered. In an AD animal model, experiments showed that this approach improved cognitive impairments by considerably suppressing neuroinflammation, lowering the burden of Aβ plaque, and promoting glial cell polarization towards an anti-inflammatory phenotype (M2).

Reza-Zaldivar et al. demonstrated in an AD mouse model that mesenchymal stem cell extracellular vesicles (MSC-EVs) improved cognitive function and increased hippocampal neurogenesis at 28 days post-treatment [138]. Mao et al. demonstrated in vitro that EVs from human umbilical cord mesenchymal stem cells modulate inflammatory cytokine levels by regulating microglial activity [98]. Injection of EVs derived from human umbilical cord mesenchymal stem cells into an AD mouse model has been demonstrated to ameliorate cognitive impairment [154].

### 4.4. Parkinson’s Disease

The gradual degradation of dopaminergic neurons in the substantia nigra pars compacta is the primary pathogenic process of Parkinson’s disease (PD) [155]. Patients experience the typical motor signs of bradykinesia, postural instability, muscle rigidity, and resting tremor when this loss surpasses 50% and decreases dopamine levels by more than 80% [156]. The causes of neuronal death are numerous and intricate, but they mostly include oxidative stress, neuroinflammation, mitochondrial dysfunction, dysregulated autophagy, and aberrant α-synuclein aggregation. These elements work together to hasten the death of neurons [157]. Even while levodopa and other modern treatment drugs are good at reducing motor symptoms, they are unable to stop ongoing neuronal loss, have trouble controlling non-motor symptoms, and can cause long-term adverse effects like drug tolerance and dyskinesias [158].

To solve this crucial problem, scientists created Neutro/miR-188-3p bio-mimetic vesicles by fusing neutrophil membranes with liposomes. Efficient BBB crossing is made possible by adhesion molecules (like L-selectin) and chemokine receptors (like CX3CR1) that are kept on the membrane surface. These molecules provide long circulation characteristics and chemotaxis towards inflammatory brain areas. The CDK5/NLRP3 signaling pathway is targeted and downregulated by the loaded miR-188-3p, which prevents autophagy and pyroptosis in dopaminergic neurons. Experiments showed that this vesicle could greatly reduce α-synuclein aggregation and dopaminergic neuronal degeneration in MPP^+^-induced cellular models and in MPTP mouse models [139]. Additionally, RVG-modified exosomes derived from primary DCs carrying shRNA-MCs or the aptamer F5R1 administered systemically could alleviate α-synuclein aggregation and dopaminergic neuron loss in PD mice [159]. This study shows the great potential of exosomes in the treatment of neurodegenerative illnesses [160]. Zhang et al. found that pathologic α-synuclein deposits may induce lysosomal dysfunction in astrocytes, which in turn might stimulate the release of increased amounts of extracellular vesicles [161]. Results show that peripheral blood contains astrocyte-derived EVs that carry α-synuclein, which could be a useful biomarker for the clinical diagnosis or differential diagnosis of PD.

## 5. Nasal–Cerebral Delivery Routes for Biomimetic Nanodrugs Modified with Cell Membranes and Vesicles

The main challenge in treating disorders of the CNS is overcoming the BBB to achieve effective brain accumulation and prolonged retention. This is true even though nanodrugs modified with cell membranes and vesicles show significant advantages in improving overall drug delivery efficiency and targeting—such as improved biocompatibility and reduced immunogenicity—thereby mitigating drug distribution in non-target tissues and enhancing stability [162]. Such medications are nonetheless limited by the BBB and dissipation in the systemic circulation when taken via traditional oral or injectable methods, which reduces their therapeutic effectiveness [163]. A significant advancement in this area is made by intranasal administration, which not only circumvents the BBB and systemic circulation by using the nasal mucosa’s special pathway for direct cerebral access, greatly increasing the effectiveness of drug delivery to the brain, but also increases patient compliance due to its painless, non-invasive administration [164]. For the long-term treatment needs of illnesses of the CNS, this makes it especially appropriate. By contrast, the best way for bionic nanomedicines that are altered for cell membranes and cell membrane-derived vesicles to reach the CNS is through intranasal administration, which increases the therapeutic potential of these formulations.

### 5.1. Physiological Structure of the Nasal Cavity

The nasal septum separates the two compartments of the human nasal cavity, a complicated structure that is 12 to 15 cm long [164]. Nasal mucus normally maintains an average pH of 5.5 to 6.5 on the mucosa that lines the inside of the nasal cavity. The vestibule, respiratory zone, and olfactory zone are the three main functional parts of the nose cavity, which can be separated based on morphological and functional distinctions [165]. The olfactory and respiratory zones of the nasal cavity are where most drug absorption occurs. The respiratory zone is the greatest functional area in the nasal cavity, with a total surface area of about 130 cm^2^. Only a small percentage of drugs taken through this area make it to the CNS; the majority enter the systemic circulation. The olfactory zone is located beneath the ethmoid plate at the upper part of the nasal cavity [166]. It is essential for direct distribution from the nasal cavity to the brain, despite having a very tiny surface area of 10%. Olfactory cells, supporting cells, and basal cells make up its epithelial layer. Bipolar neurons called olfactory cells have dendrites that reach into the mucosal layer and axons that cross the basement membrane to meet inside the olfactory nerve before entering the CNS’s olfactory bulb [167].

### 5.2. Nasal–Cerebral Delivery Routes

#### 5.2.1. Direct Pathway

**Figure 3:**
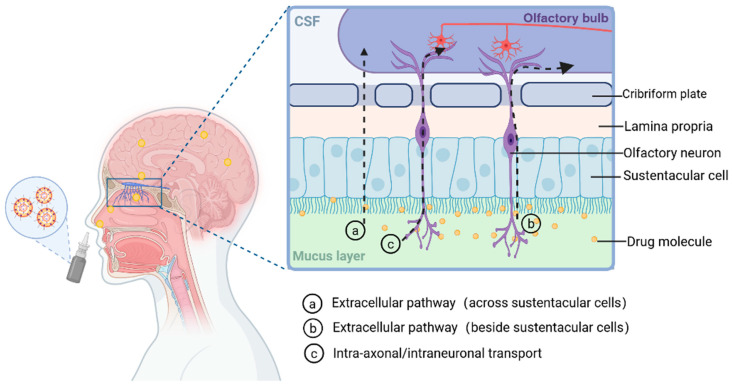
Nasal-brain drug delivery routes via the olfactory system. (**a**) Extracellular pathway (across sustentacular cells): Drugs pass through sustentacular cells, traverse the nasal epithelium to reach the lamina propria, and are then transported toward the olfactory bulb and CSF. (**b**) Extracellular pathway (beside sustentacular cells): Drugs diffuse along the paracellular spaces beside sustentacular cells, enter the lamina propria, and are then transported toward the olfactory bulb. (**c**) Intra-axonal/intraneuronal transport: Drugs are internalized by olfactory neurons, transported along axons to the olfactory bulb, and then released into brain regions (Created in BioRender, Ding, F (2025) https://BioRender.com/99krrc4).

One important avenue for direct medication administration to the brain is the olfactory system. Drugs can enter the CNS directly and avoid systemic circulation thanks to the direct connection it creates between the olfactory nerves in the upper nasal cavity and the olfactory bulb in the brain (Figure 3). The olfactory tract, anterior olfactory nuclei, piriform cortex, entorhinal cortex, and amygdala are among the brain regions to which sensory fibers from the olfactory bulb are connected [168]. Drugs entering the brain via the olfactory pathway primarily operate through two mechanisms [169]: (1) Intra-axonal/intraneuronal transport: Drugs can be internalized by olfactory sensory neurons through endocytosis or pinocytosis and then transported along their axons to the olfactory bulb, after which they are released into different regions of the brain via exocytosis [170]. This intraneuronal transport is considered a relatively slow transport mechanism, taking several hours to several days. (2) Extracellular pathway: This pathway starts with drugs passing through the nasal epithelium to reach the lamina propria, then traversing the olfactory mucosa either across sustentacular cells or along the paracellular spaces beside sustentacular cells, and subsequently continuing to be transported along the periphery of neuronal axons and blood vessels [171]. Compared with intra-axonal transport, this is a much faster and higher-flux process. Drugs reach the olfactory bulb and the CNS within 1.5–6 h after entry into the circulation to complete the process [172]. Therefore, extracellular transport is considered to be the fast-transporting process to load drugs into the brain.

The terminals of the trigeminal nerve in the nasal respiratory region are the source of the trigeminal nerve route [173]. The extracellular pathway refers to the drug permeating through the perineural space, which is the loose connective tissue surrounding the trigeminal nerve, until it reaches the base of the cranium. They eventually arrive at the trigeminal ganglion and trigeminal nucleus in the brainstem after passing through the foramen rotundum, foramen ovale, or superior orbital fissure [174]. Particles may also be transported transcellularly via Schwann cell endocytosis and then anterogradely along axonal microtubules, provided they are tiny enough and have had their surfaces altered with transmembrane peptides. Drug entry into the caudate and rostrum areas of the brain is facilitated by the trigeminal nerve innervating the nasal cavity entering the brainstem through the pons and the forebrain through the cribriform plate. This constitutes a primary focus for intra-cerebral (IN) drug delivery [175].

#### 5.2.2. Indirect Pathways

A proportion of intranasally administered drugs, particularly those distributed in the respiratory zone, can be absorbed by the respiratory epithelium into the systemic circulation [176]. Due to the selective nature of the BBB, small molecules are able to cross it easily, whereas others have decreased efficiency of transport into the brain via the bloodstream.

A new route for drugs from the nose to the brain is the lymphatic system. The drug that is administered intranasally enters the nasal lymphatic vessels and reaches the brain through the perivascular space or CSF exchange [177]. As the primary structure connecting the peripheral lymphatic system and CSF, the cribriform plate is very important for this process. Before reaching the cervical lymph nodes via the drainage of nasal lymphatic arteries [178], CSF first goes through the ethmoid labyrinth and the areas surrounding the olfactory sense neurons. It has been found that meningeal lymphatic vessels (MLVs) are substantially large channels that may provide BBB circumvention. When it is necessary to pass a biologic or macromolecule that is prevented from crossing the blood–brain barrier, this method is particularly useful.

## 6. Application of Cell Membrane- and Vesicle-Based Bionic Nanodrugs in the Nasoencephalic Pathway

Intranasal delivery has emerged as a significant route for treating CNS disorders and has received Food and Drug Administration (FDA) approval for a range of CNS-related emergency and acute therapies [179]. Currently approved formulations explicitly leveraging the nasal-brain pathway include esketamine for treatment-resistant depression; midazolam and diazepam for acute epileptic seizures; and naloxone for opioid overdose rescue [180]. However, existing clinical formulations are predominantly simple solutions or traditional preparations, primarily relying on the drug’s lipophilicity and high-concentration administration. They still face core limitations in traversing various biological barriers. To overcome this issue, the following sections will focus on exploring application cases of BDDS based on cell membranes and vesicles in CNS diseases.

### 6.1. Application of Cell Membranes and Membrane Vesicles in Stroke Therapy

**Figure 4:**
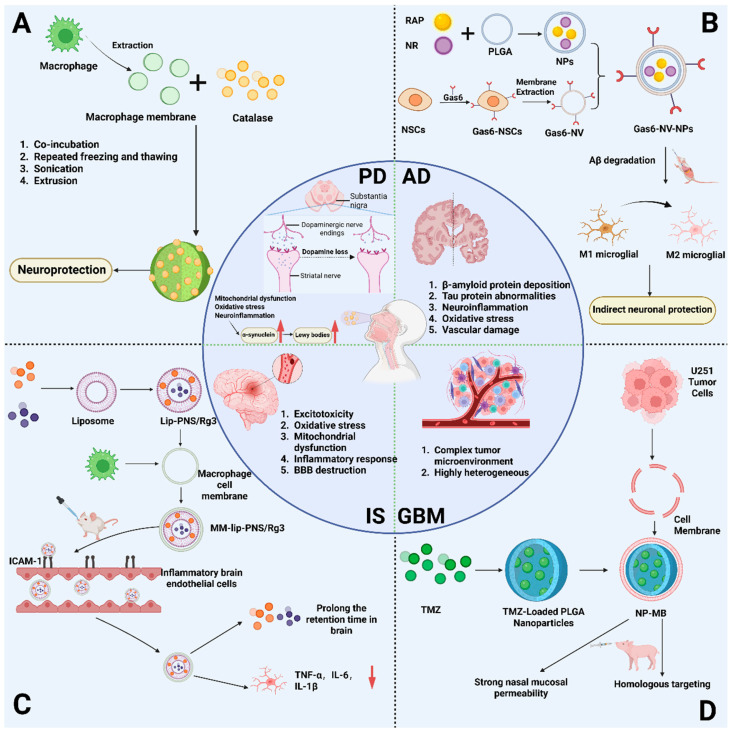
Application of cell membrane- and vesicle-based biomimetic nanodrugs in the nasoencephalic pathway for treating various neurological diseases. (**A**) Catalase-loaded exosomes, after intranasal administration, are internalized by neurons and microglia in PD mice, exerting significant neuroprotective effects. (**B**) The Gas6-NV-NPs system is formed by modifying poly(lactic-co-glycolic acid) (PLGA) nanoparticles co-encapsulating rapamycin (RAP) and nicotinamide riboside (NR) with membranes from neural stem cells overexpressing glial cell line-derived neurotrophic factor (GDNF). This system targets pathological regions of AD, not only enhancing the phagocytic clearance of β-amyloid (Aβ) by microglia but also regulating microglia to an anti-inflammatory phenotype, achieving brain targeting and neuronal repair. (**C**) Macrophage membrane-coated liposomes (MM-Lip-Rg3/PNS) loaded with ginsenoside Rg3 and total saponins, by virtue of the affinity of macrophage membrane proteins for inflamed cerebral microvascular endothelial cells, cross the BBB to exert neuroprotective effects, addressing inflammation and tissue damage after ischemia. (**D**) Poly(lactic-co-glycolic acid) (PLGA) nanoparticles loaded with temozolomide (TMZ) and coated with U251 glioma cell membranes (NP-MB), combined with the nasoencephalic drug delivery pathway, enhance the targeting to GBM cells, thereby achieving effective treatment (Created in BioRender, Ding, F (2025) https://BioRender.com/h1hyqty).

In stroke, researchers aimed to leverage the natural affinity of macrophage membrane proteins for inflamed cerebral microvascular endothelial cells to overcome the limitations of therapeutic drugs failing to accumulate at the ischemic site (due to the lack of BBB) and post-ischemic inflammation enhancing the damage [181]. They made macrophage membrane-coated liposomes (MM-Lip-Rg3/PNS) loaded with ginsenoside Rg3 and total saponins from Panax notoginseng (PNS) using a co-extrusion technique, as shown in Figure 4C. By actively binding and navigating inflammatory endothelium cells through direct or indirect nasal-brain pathways, this nanodelivery technology uses intranasal dosing to target microglia and ischemic regions. This method not only eliminates the problem of drug penetration across the BBB but it also greatly improves drug accumulation and half-life within the brain. By fully leveraging the innate targeting properties of macrophages, it offers a novel, highly efficient, and precise therapeutic approach for IS [182]. Furthermore, SC-EVs (ProtheraCytes) have also demonstrated positive effects. ProtheraCytes constitute a non-adherent CD34+ cell population derived from human peripheral blood and umbilical cord blood. A research team revealed that these cells secrete growth factors and EVs associated with angiogenesis and vasogenesis. Data indicate that intranasal transplantation of ProtheraCytes three days post-stroke in adult rats reduces stroke-induced behavioral deficits and histological damage. Furthermore, upregulation of human CD63+ EVs was detected in the ischemic brains of stroke animals receiving ProtheraCytes, correlating with increased levels of DCX-labelled neurogenesis, VEGFR1-associated angiogenesis and vasculogenesis, and reduced Iba1-labelled inflammation. Additionally, recognition and binding of CD63 and apolipoprotein ApoA-1 enhanced EV secretion [183].

### 6.2. Recent Advances in Engineered Cell Membranes and Exosomes for AD Therapy

Regarding AD, multiple studies have focused on developing nasal-brain delivery systems for nanomedicines. Among these, a system based on engineered neural stem cell membrane modification is particularly representative. This system employs PLGA as a carrier, co-encapsulating rapamycin (RAP) and nicotinamide riboside (NR) to form nanoparticles (NPs) [184] (Figure 4B). These are then modified using membranes from glial cell line-derived neurotrophic factor (GDNF)-overexpressing neural stem cells, constructing the Gas6-NV-NPs composite system. The core advantage of this system lies in its precise targeting of pathological regions in AD through the specific binding of Gas6 protein to surface receptors on microglia. This not only enhances microglia’s phagocytic clearance of Aβ but also modulates their anti-inflammatory phenotype. In AD mouse models, this system demonstrated favorable brain targeting and neuronal repair effects, offering novel insights for AD nanotherapy.

In recent years, intranasal administration of stem cell-derived exosomes has garnered increasing attention. A research team carried out the first clinical trial of intranasal exosome therapy derived from mesenchymal stem cells for AD. The results showed that the treatment was safe and effective, effective as some existing drugs or better, bringing new expectations for Alzheimer’s patients [185]. Thereafter, in the following year, in the study by Madhu et al., the authors demonstrated that a nasal spray consisting of EVs derived from neural stem cells could reduce inflammation and plaque in the brain and extend cognitive function by 10–15 years, which would delay the progression of AD [186].

### 6.3. Potential of Exosomes in Nasoencephalic Delivery for PD

So far, polymeric and lipid nanoparticles are the main research focus of nasoencephalic delivery for PD [187]. However, studies on using exosomes in nasoencephalic delivery have also been attracting more and more attention recently. Both naturally occurring and engineered EVs can be used to deliver various therapeutic molecules like antioxidants, growth factors, and siRNA through different administration routes for PD treatment. SHEDs represent a unique subtype of mesenchymal stem cells with special neurogenic properties because they can differentiate into DA-neuron like cells and Schwann cells. Narbute et al. managed to transfer EVs derived from shed dental stem cells (SHEDs) to the brain through the intranasal route in a rat model [188]. It was found that EVs could improve 6-hydroxydopamine (6-OHDA)-induced motor deficits, expressed as improved gait and decreased contralateral turning. Haney et al. tried to load catalase into RAW264.7 cell exosomes using multiple approaches to obtain loaded exosomes, including co-incubation, repeated freeze–thaw cycles, sonication, or extrusion [189] (Figure 4A). In vivo studies demonstrated that following intranasal administration, these catalase-loaded exosomes were successfully internalized by neurons and microglia in PD mice, exhibiting significant neuroprotective effects.

### 6.4. Application of Bionic Cell Membranes in GBM

Nanodrug delivery systems engineered based on cancer cell membrane modifications have become a prevalent strategy for the transnasal treatment of GBM. Researchers have developed a novel combined therapeutic strategy for GBM, comprising temozolomide (TMZ)-loaded PLGA nanoparticles encapsulated with U251 glioma cell membranes (NP-MB) [190] (Figure 4D). This study comprehensively characterized the NP-MB nanoplatform and evaluated its potential for drug release and penetration patterns. Through in vitro, ex vivo, and in vivo experiments, the team demonstrated that NP-MB achieves ‘homotypic recognition’ via homologous cell membranes, significantly enhancing targeting to GBM cells. Combined with a nasal-brain delivery route, this platform offers an efficient and novel solution for GBM treatment.

### 6.5. Bacterial Exosomes: Emerging Vectors for Naso-Cerebral Delivery

Whilst the aforementioned studies predominantly focus on stem cell-derived biomimetic nanomedicines, recent advances in this field provide substantial evidence for naso-cerebral delivery via BEVs. Researchers discovered that Lactobacillus plantarum (Lp) serves as an effective nasal drug delivery vehicle [191]. The surface oligopeptide substrate-binding protein (OppA) on this strain specifically binds to N-acetylsulphate acetyl heparan (NaSH) in the olfactory epithelium (OE), enabling efficient delivery within the olfactory epithelium. Subsequently, the payload molecules released by the strain are transported and accumulate within the brain. This discovery leads to a significant hypothesis: if intact bacteria can serve as delivery vehicles for the nose-to-brain pathway via their specific molecules, then the nanoscale vesicles secreted by bacteria—bacterial extracellular vesicles—possess equally immense potential. The innate ability of BEVs to cross the BBB, combined with their nanoscale dimensions, enables efficient uptake by the olfactory epithelium and entry into the brain via the olfactory pathway, delivering therapeutic payloads directly to the lesion site. Through engineered modifications, we can further enhance their targeting capabilities and improve therapeutic efficacy. Consequently, intranasal delivery of BEVs holds promise as a novel and highly effective strategy for treating neurological disorders.

## 7. Advantages and Challenges of Bionic Drug Delivery Systems Based on Cell Membrane and Vesicle in Nasoencephalic Delivery

### 7.1. Core Advantages in Nasoencephalic Delivery

The cell membrane’s core advantage lies in its high biological compatibility and immune evasion capability. Its surface native molecules significantly reduce immune clearance, prolonging nasal retention time and providing ample time for drug transport [192]. Concurrently, cell membranes possess customizable targeting modification potential. By conjugating homing peptides, they enable ‘dual-stage targeting’ to the ischemic penumbra or inflammatory foci, elevating drug concentration enrichment within the brain [193]. The advantages of EVs lie in their exceptional barrier-crossing capabilities and efficient delivery of bioactive molecules [194]. Their small particle size (30–150 nm) closely matches the intercellular gaps of the nasal olfactory epithelium, enabling rapid entry into the olfactory bulb via perineural spaces [183]. Natural surface proteins like TfR and LFA-1 on EVs mediate BBB crossing [195]. As an emerging delivery vehicle, BEVs’ core advantages lie in their efficient mucosal adhesion and penetration capabilities, coupled with low-cost, scalable production potential [196].

### 7.2. Challenges in Nasal–Cerebral Delivery

All carriers face rapid clearance by the nasal mucociliary clearance system [197]. Proteases and nucleases within the nasal cavity further degrade targeted molecules and drugs, severely impairing delivery efficiency. Even upon reaching the brain, carriers remain constrained by physical barriers within pathological microenvironments [198], hindering uniform distribution. Furthermore, LPS components in allogeneic cell membranes and BEVs may trigger immune rejection or inflammatory responses, while long-term toxicity data remain scarce, requiring further extended animal studies to assess potential risks from intracerebral accumulation [199].

## 8. Conclusions and Future Prospects

In summary, biomimetic nanodrug delivery systems based on cell membranes and vesicles exhibit significant and undeniable potential in the field of nose-to-brain delivery for the treatment of CNS diseases. By cleverly combining the biological characteristics of natural cell membranes or vesicles with the characteristics of nanocarriers, in addition to successfully bypassing the BBB, which has been a longstanding key technical problem in drug transportation to the CNS, this method also increases the enrichment concentration of drugs at the lesion through fine control of targeting and decreases the drug distribution in non-target tissues. Ultimately, it enhances the treatment effect and alleviates toxic side effects.

In terms of different carriers, cell membrane modified nanocarriers of different types exhibit high precision in the treatment of specific CNS diseases due to their surface characteristics and biological advantages. EVs provide an effective approach for endogenous therapy, leveraging their excellent barrier-crossing capabilities and ability to deliver bioactive molecules. BEVs, on the other hand, exhibit distinctive strengths in large-scale production and nasal mucosa adhesion and penetration. In the application of nose-to-brain delivery pathways, these biomimetic nanodrug delivery systems can fully utilize the physiological structure of the nasal cavity and delivery mechanisms to further improve the efficiency of drug entry into the brain. They thus offer novel and effective strategies for the treatment of various intractable CNS diseases, such as GBM, IS, AD, and PD.

Nevertheless, the field currently faces numerous urgent challenges to be addressed. During nose-to-brain delivery, the rapid clearance by the nasal mucociliary system, the degradation of drugs and targeting molecules by enzymes in the nasal cavity, the distribution issues of drugs in the pathological microenvironment after entering the brain, and potential toxicity risks all compromise delivery efficiency and safety. Therefore, we should perhaps pay attention to hydrogel and other carriers’ application because hydrogels have mucosal adhesion properties and can provide a protective enzyme barrier. Meanwhile, each type of carrier also has specific shortcomings in extraction, production, and immunogenicity. Furthermore, in clinical translation, membrane separation methods such as differential centrifugation and ultrasonic disruption can lead to low purity and unstable results [60]. Membrane extraction techniques employed during preparation, including hypotonic swelling and ultrasonic disruption, may damage membrane proteins or alter membrane composition. Regarding scale-up production, current processes are predominantly laboratory-scale. During scaling, parameters like pressure and flow rate in co-extrusion techniques are difficult to control, leading to unstable particle size distribution, inconsistent membrane coating efficiency, and poor batch-to-batch reproducibility [200]. Simultaneously, the large-scale, stable acquisition of high-quality, uniform cell membranes remains challenging, limiting mass production feasibility. Future research should systematically advance the development of biomimetic nanodrug delivery systems based on cell membranes and vesicles to overcome the bottlenecks in their clinical translation for nose-to-brain delivery in CNS diseases.

## Figures and Tables

**Table 1:** Advantages, drawbacks, and applications of different types of cell membranes.

Cell Membrane Type	Core Biological Advantages	Potential Applications in CNS Diseases	Main Drawbacks
Red Blood Cell Membrane (RBC Membrane)	Immune Evasion and Long Circulation	As a long-acting drug delivery platform, it achieves targeted delivery to brain lesions through surface modification [28,29,30].	Surface modification may induce hemolysis [53].
Platelet Membrane	Natural Targeting Ability	For thrombus-targeted therapy of IS, drugs are accurately delivered to the lesion site [35].	The cycle time is shorter than that of the red blood cell membrane [54].
Macrophage Membrane	Inflammatory Targeting and Immune Regulation	It treats neuroinflammation-related diseases to achieve precise localization of lesions and immune regulation [38,39].	Weak active targeting capability [55].
Neutrophil Membrane	Chemotactic Migration Ability	It treats acute or chronic neuroinflammation and delivers drugs efficiently to the inflammatory area [41,43].	Organ toxicity [56].
Stem Cell Membrane	BBB Crossing and Precise Homing	It treats neurodegenerative diseases, improving the efficiency of drug entry into the brain and the enrichment degree at the lesion site [50,52].	Low specificity [53].

**Table 2:** Comparison of Characteristics of Three Biological Carriers.

	Cell Membrane-Modified Nanocarriers	Extracellular Vesicles (EVs)	Bacterial Extracellular Vesicles (BEVs)
Nature	Artificially camouflaged core–shell structure	Naturally occurring carriers secreted by cells	Naturally occurring carriers secreted by bacteria
Source	Membranes extracted from intact cells [20]	Vesicles secreted by living eukaryotic cells [69]	Shed from or secreted by bacterial outer membranes [114]
Preparation	Physical disruption and fusion with high controllability [24]	Cell culture and purification with limited yield	Bacterial culture and purification, enabling easy large-scale production [123]
Advantages	Adjustable nanocores with diverse functions	Carry endogenous molecules with inherent biological activity [79]	Possess pathogen-associated molecular patterns (PAMPs) on the surface, providing efficient immunostimulation
Limitations	Complex preparation process, which may damage membrane proteins [60]	Difficulty in standardizing yield and purity [102]	Potential toxicity, requiring detoxification or modification [122]

**Table 3:** Application of Various Biomimetic Materials in Different Neurological Diseases.

Type of Disease	Biomimetic Material	Critical Role	Specific Mechanism
Glioblastoma Multiforme (GBM)	Red Blood Cell Membrane-Mimetic Nanoparticles (Ang-RBCm-CA/siRNA)	Long Circulation and Active Targeting	They utilize the red blood cell membrane to prolong circulation, and after modification with Angiopep-2, they can cross the BBB and target glioma [127].
	Macrophage Membrane-Mimetic Nanocarriers (PD-1-MM@PLGA/RAPA)	BBB Penetration and Tumor Accumulation	Engineered macrophage membranes can assist nanoparticles in effectively penetrating the BBB and accumulating in tumor tissues [128].
	M1 Macrophage-Derived Extracellular Vesicles (M1EVs)	Chemotaxis and Immunomodulation	They utilize the chemotactic properties of M1 macrophages to accumulate at the tumor site and regulate immune cells in the tumor microenvironment [129].
	Bacterial Outer Membrane Vesicles (OMVs)	Multifunctionality	They act as a carrier and immune adjuvant, can cross the BBB, and simultaneously enhance antitumor efficacy through photothermal effects and immune activation [130].
Ischemic Stroke (IS)	Red Blood Cell Membrane Nanoparticles (SHp-RBC-NPs)	Targeting and ROS-Responsive Release	They use red blood cell membranes for encapsulation and are modified with homing peptides to achieve precise targeting of ischemic brain tissue and effectively release drugs based on the ROS microenvironment of the lesion [131].
	Platelet Membrane Nanocarriers (APLT-PA)	Thrombus Targeting and Bleeding Risk Reduction	They leverage the natural targeting properties of platelet membranes to precisely deliver the thrombolytic drug tPA to the thrombus site [132].
	Neutrophil Membrane Nanoparticles (SNM-NPs)	Inflammatory Chemotaxis and Oxidative Stress Response	They utilize the chemotaxis of neutrophil membranes and the dual targeting of homing peptides to guide nanoparticles to migrate to the injured area and effectively release drugs in response to ROS [133].
	Bacterial Outer Membrane Vesicles (OMV@PGZ)	Brain Entry and Dual Pathway Inhibition	They utilize the interaction between OMVs and neutrophils to efficiently deliver drugs across the BBB and simultaneously inhibit the inflammatory and ferroptosis pathways [134].
	Lactobacillus plantarum-Derived Extracellular Vesicles (LEVs)	Neurorepair	They regulate downstream signaling pathways by upregulating miRNAs, improve neuronal apoptosis, and promote neural repair [135].
Alzheimer’s Disease (AD)	Engineered Macrophage Membrane Nanocarriers (OT-Lipo@M)	BBB Crossing and Inflammatory Targeting	They utilize the phagocytosis resistance and pro-inflammatory tropism of macrophage membranes to actively cross the BBB and target inflammatory foci in the AD brain [136].
	Neutrophil Membrane Nanoparticles (Neu-MOF/Fla)	BBB Crossing and Multifunctional Synergy	They cross the BBB by utilizing the chemotaxis of neutrophil membranes, while the nanozyme scavenges ROS and releases anti-inflammatory factors to achieve combined therapy [137].
	Mesenchymal Stem Cell-Derived Extracellular Vesicles (MSC-EVs)	Endogenous Therapy	The EVs themselves possess the capabilities of immunomodulation, neuroprotection, and promotion of neurogenesis [138].
Parkinson’s Disease (PD)	Neutrophil Membrane-Mimetic Vesicles (Neutro/miR-188-3p)	BBB Crossing and Gene Therapy	They efficiently cross the BBB by utilizing the chemotaxis of neutrophil membranes, release miRNAs to regulate specific signaling pathways, and inhibit neuronal apoptosis [139].

## Data Availability

Data availability is not applicable to this article as no new data were created or analyzed in this study.

## References

[1] Wang A.Y.L., Kao H.K., Liu Y.Y., Loh C.Y.Y. (2025). Engineered extracellular vesicles derived from pluripotent stem cells: A cell-free approach to regenerative medicine. Burn. Trauma.

[2] GBD 2021 Nervous System Disorders Collaborators (2024). Correction: Global, regional, and national burden of disorders affecting the nervous system, 1990–2021: A systematic analysis for the Global Burden of Disease Study 2021. Lancet Neurol..

[3] Li J., Wei Y., Zhang C., Bi R., Qiu Y., Li Y., Hu B. (2023). Cell-Membrane-Coated Nanoparticles for Targeted Drug Delivery to the Brain for the Treatment of Neurological Diseases. Pharmaceutics.

[4] Pandya J.D., Musyaju S., Modi H.R., Okada-Rising S.L., Bailey Z.S., Scultetus A.H., Shear D.A. (2024). Intranasal delivery of mitochondria targeted neuroprotective compounds for traumatic brain injury: Screening based on pharmacological and physiological properties. J. Transl. Med..

[5] Abdelsalam M., Ahmed M., Osaid Z., Hamoudi R., Harati R. (2023). Insights into Exosome Transport through the Blood–Brain Barrier and the Potential Therapeutical Applications in Brain Diseases. Pharmaceuticals.

[6] Choi H.K., Chen M., Goldston L.L., Lee K.B. (2024). Extracellular vesicles as nanotheranostic platforms for targeted neurological disorder interventions. Nano Converg..

[7] Ezike T.C., Okpala U.S., Onoja U.L., Nwike C.P., Ezeako E.C., Okpara O.J., Okoroafor C.C., Eze S.C., Kalu O.L., Odoh E.C. (2023). Advances in drug delivery systems, challenges and future directions. Heliyon.

[8] Yi L.X., Tan E.K., Zhou Z.D. (2024). Passive immunotherapy for Alzheimer’s disease: Challenges & future directions. J. Transl. Med..

[9] Liang Y., Iqbal Z., Lu J., Wang J., Zhang H., Chen X., Duan L., Xia J. (2023). Cell-derived nanovesicle-mediated drug delivery to the brain: Principles and strategies for vesicle engineering. Mol. Ther..

[10] Cunha S., Almeida H., Amaral M.H., Lobo J.M.S., Silva A.C. (2017). Intranasal lipid nanoparticles for the treatment of neurodegenerative diseases. Curr. Pharm. Des..

[11] Cunha S., Amaral M.H., Lobo J.M.S., Silva A.C. (2017). Lipid Nanoparticles for Nasal/Intranasal Drug Delivery. Crit. Rev. Ther. Drug Carr. Syst..

[12] Lochhead J.J., Thorne R.G. (2012). Intranasal delivery of biologics to the central nervous system. Adv. Drug Deliv. Rev..

[13] Shen J., Duan X., Xie T., Zhang X., Cai Y., Pan J., Zhang X., Sun X. (2025). Advances in locally administered nucleic acid therapeutics. Bioact. Mater..

[14] Islam S.U., Shehzad A., Ahmed M.B., Lee Y.S. (2020). Intranasal Delivery of Nanoformulations: A Potential Way of Treatment for Neurological Disorders. Molecules.

[15] Liu Y., Guo Q., Shi Y., Cui M., Jing F. (2024). Research progress of novel anti-tumor drug formulations. Front. Oncol..

[16] Wu D.D., Salah Y.A., Ngowi E.E., Zhang Y.X., Khattak S., Khan N.H., Wang Y., Li T., Guo Z.H., Wang Y.M. (2023). Nanotechnology prospects in brain therapeutics concerning gene-targeting and nose-to-brain administration. iScience.

[17] Sun J., Han Y., Dong J., Lv S., Zhang R. (2023). Melanin/melanin-like nanoparticles: As a naturally active platform for imaging-guided disease therapy. Mater. Today Bio.

[18] Zhou J., Jiang Z., Sun R., Pan D., Du Q., Zhou X., Chen Y., Chen Y., Peng J. (2024). Comparison of cell delivery and cell membrane camouflaged PLGA nanoparticles in the delivery of shikonin for colorectal cancer treatment. Colloids Surf. B Biointerfaces.

[19] Zhong Z., Deng W., Wu J., Shang H., Tong Y., He Y., Huang Q., Ba X., Chen Z., Tang K. (2024). Cell membrane coated nanoparticles as a biomimetic drug delivery platform for enhancing cancer immunotherapy. Nanoscale.

[20] Zhou Y., Wang X., Tian X., Zhang D., Cui H., Du W., Yang Z., Li J., Li W., Xu J. (2025). Stealth missiles with precision guidance: A novel multifunctional nano-drug delivery system based on biomimetic cell membrane coating technology. Mater. Today Bio.

[21] Liu Y., Li J., Guo H., Fang C., Yang Q., Qin W., Wang H., Xian Y., Yan X., Yin B. (2024). Nanomaterials for stroke diagnosis and treatment. iScience.

[22] Hao C., Sha M., Ye Y., Wang C. (2023). Cell Membrane-Derived Nanovehicles for Targeted Therapy of Ischemic Stroke: From Construction to Application. Pharmaceutics.

[23] Skalickova M., Hadrava Vanova K., Uher O., Leischner Fialova J., Petrlakova K., Masarik M., Kejík Z., Martasek P., Pacak K., Jakubek M. (2024). Injecting hope: The potential of intratumoral immunotherapy for locally advanced and metastatic cancer. Front. Immunol..

[24] Liao W., Lu Z., Wang C., Zhu X., Yang Y., Zhou Y., Gong P. (2024). Application and advances of biomimetic membrane materials in central nervous system disorders. J. Nanobiotechnol..

[25] Lin D., Yang H., Liang X., Yang M., Zhao Y. (2025). The involvement of mitochondria in erythrocyte pathology and diseases: From mechanisms to therapeutic strategies. Clin. Exp. Med..

[26] Xie X., Wang H., Williams G.R., Yang Y., Zheng Y., Wu J., Zhu L.M. (2019). Erythrocyte Membrane Cloaked Curcumin-Loaded Nanoparticles for Enhanced Chemotherapy. Pharmaceutics.

[27] Xu X., Li T., Jin K. (2022). Bioinspired and Biomimetic Nanomedicines for Targeted Cancer Therapy. Pharmaceutics.

[28] Lu H., Jiang Y., Luo R., Zhou D., Zheng F., Shi L., Zhang H., Wang Y., Xu X., Zou R. (2025). Engineered hybrid cell membrane nanosystems for treating cardiovascular diseases. Mater. Today Bio.

[29] Sun X., Wang C., Gao M., Hu A., Liu Z. (2015). Remotely Controlled Red Blood Cell Carriers for Cancer Targeting and Near-Infrared Light-Triggered Drug Release in Combined Photothermal–Chemotherapy. Adv. Funct. Mater..

[30] Hu C.-M.J., Zhang L., Aryal S., Cheung C., Fang R.H., Zhang L. (2011). Erythrocyte membrane-camouflaged polymeric nanoparticles as a biomimetic delivery platform. Proc. Natl. Acad. Sci. USA.

[31] Li T., Qin X., Li Y., Shen X., Li S., Yang H., Wu C., Zheng C., Zhu J., You F. (2020). Cell Membrane Coated-Biomimetic Nanoplatforms Toward Cancer Theranostics. Front. Bioeng. Biotechnol..

[32] Burnouf T., Walker T.L. (2022). The multifaceted role of platelets in mediating brain function. Blood.

[33] Liu W.S., Wu L.L., Chen C.M., Zheng H., Gao J., Lu Z.M., Li M. (2023). Lipid-hybrid cell-derived biomimetic functional materials: A state-of-the-art multifunctional weapon against tumors. Mater. Today Bio.

[34] Avgoustakis K., Angelopoulou A. (2024). Biomaterial-Based Responsive Nanomedicines for Targeting Solid Tumor Microenvironments. Pharmaceutics.

[35] Li M., Li J., Chen J., Liu Y., Cheng X., Yang F., Gu N. (2020). Platelet Membrane Biomimetic Magnetic Nanocarriers for Targeted Delivery and in Situ Generation of Nitric Oxide in Early Ischemic Stroke. ACS Nano.

[36] Du Y., Yang Y., Zheng B., Zhang Q., Zhou S., Zhao L. (2025). Finding a needle in a haystack: Functional screening for novel targets in cancer immunology and immunotherapies. Oncogene.

[37] Garmendia Urdalleta A., Van Poll M., Fahy N., Witte-Bouma J., Van Wamel W., Apachitei I., Zadpoor A.A., Fratila-Apachitei L.E., Farrell E. (2023). The response of human macrophages to 3D printed titanium antibacterial implants does not affect the osteogenic differentiation of hMSCs. Front. Bioeng. Biotechnol..

[38] Stewart A.G., Beart P.M. (2016). Inflammation: Maladies, models, mechanisms and molecules. Br. J. Pharmacol..

[39] Liu B., Yan W., Luo L., Wu S., Wang Y., Zhong Y., Tang D., Maruf A., Yan M., Zhang K. (2021). Macrophage membrane camouflaged reactive oxygen species responsive nanomedicine for efficiently inhibiting the vascular intimal hyperplasia. J. Nanobiotechnol..

[40] Liu W., Zou Z., Li W., Yang G., Zhang J., Zhang Z., Yao H. (2025). Research status and future perspectives of IL-27 in the treatment of stroke (Review). Int. J. Mol. Med..

[41] Kallen V., Scherder R., Cramer M.J., Stam J., Johnson B., Scherder E. (2021). Neutralizing a Springboard for Inflammation: Physical Activity to Control the Immune Network. Healthcare.

[42] Hao J., Chen J., Wang M., Zhao J., Wang J., Wang X., Li Y., Tang H. (2020). Neutrophils, as “Trojan horses”, participate in the delivery of therapeutical PLGA nanoparticles into a tumor based on the chemotactic effect. Drug Deliv..

[43] Wu J., Zhang Y., Chen W., Hao T., Ran C., Zhou Y., Shen Y., You W., Wang T. (2025). Smart Thrombosis Care: The Rise of Closed-Loop Diagnosis-to-Treatment Nano Systems. Int. J. Nanomed..

[44] Han D., Wang F., Qiao Z., Wang B., Zhang Y., Jiang Q., Liu M., Zhuang Y., An Q., Bai Y. (2023). Neutrophil membrane-camouflaged nanoparticles alleviate inflammation and promote angiogenesis in ischemic myocardial injury. Bioact. Mater..

[45] Liu Y., He M., Yuan Y., Nie C., Wei K., Zhang T., Chen T., Chu X. (2023). Neutrophil-Membrane-Coated Biomineralized Metal-Organic Framework Nanoparticles for Atherosclerosis Treatment by Targeting Gene Silencing. ACS Nano.

[46] Zhang Q., Dehaini D., Zhang Y., Zhou J., Chen X., Zhang L., Fang R.H., Gao W., Zhang L. (2018). Neutrophil membrane-coated nanoparticles inhibit synovial inflammation and alleviate joint damage in inflammatory arthritis. Nat. Nanotechnol..

[47] Deng S., Nie D., Huang Y., Yang Y., Liu Q., Sun Z., Jiang Q., Ling Y., Wen Y., Qu J. (2024). A Magnetic-Responsive Biomimetic Nanosystem Coated with Glioma Stem Cell Membranes Effectively Targets and Eliminates Malignant Gliomas. Biomater. Res..

[48] Fan D., Cao Y., Cao M., Wang Y., Cao Y., Gong T. (2023). Nanomedicine in cancer therapy. Signal Transduct. Target. Ther..

[49] Sipos F., Műzes G. (2022). Disagreements in the therapeutic use of mesenchymal stem cell-derived secretome. World J. Stem Cells.

[50] Choi A., Javius-Jones K., Hong S., Park H. (2023). Cell-Based Drug Delivery Systems with Innate Homing Capability as a Novel Nanocarrier Platform. Int. J. Nanomed..

[51] Ma J., Zhang S., Liu J., Liu F., Du F., Li M., Chen A.T., Bao Y., Suh H.W., Avery J. (2019). Targeted Drug Delivery to Stroke via Chemotactic Recruitment of Nanoparticles Coated with Membrane of Engineered Neural Stem Cells. Small.

[52] Kaur J., Thakran A., Naqvi S. (2025). Recent advances in cell membrane-based biomimetic delivery systems for Parkinson’s disease: Perspectives and challenges. Asian J. Pharm. Sci..

[53] Liu Y., Luo J., Chen X., Liu W., Chen T. (2019). Cell Membrane Coating Technology: A Promising Strategy for Biomedical Applications. Nanomicro Lett..

[54] Wang S., Wang R., Meng N., Guo H., Wu S., Wang X., Li J., Wang H., Jiang K., Xie C. (2020). Platelet membrane-functionalized nanoparticles with improved targeting ability and lower hemorrhagic risk for thrombolysis therapy. J. Control. Release.

[55] Gao C., Huang Q., Liu C., Kwong C.H.T., Yue L., Wan J.B., Lee S.M.Y., Wang R. (2020). Treatment of atherosclerosis by macrophage-biomimetic nanoparticles via targeted pharmacotherapy and sequestration of proinflammatory cytokines. Nat. Commun..

[56] Wang H., Zang J., Zhao Z., Zhang Q., Chen S. (2021). The Advances of Neutrophil-Derived Effective Drug Delivery Systems: A Key Review of Managing Tumors and Inflammation. Int. J. Nanomed..

[57] Zhao X., Chen W., Wu J., Shen Y., Xu B., Chen Z., Sun Y. (2025). Application of Biomimetic Cell Membrane-Coated Nanocarriers in Cardiovascular Diseases. Int. J. Nanomed..

[58] Maia R.F., Vaziri A.S., Shahbazi M.A., Santos H.A. (2025). Artificial cells and biomimicry cells: A rising star in the fight against cancer. Mater. Today Bio.

[59] Yuan S., Hu D., Gao D., Butch C.J., Wang Y., Zheng H., Sheng Z. (2025). Recent advances of engineering cell membranes for nanomedicine delivery across the blood–brain barrier. J. Nanobiotechnol..

[60] Li W., Cheng J., He F., Zhang P., Zhang N., Wang J., Song Q., Hou Y., Gan Z. (2023). Cell membrane-based nanomaterials for theranostics of central nervous system diseases. J. Nanobiotechnol..

[61] Chugh V., Vijaya Krishna K., Pandit A. (2021). Cell Membrane-Coated Mimics: A Methodological Approach for Fabrication, Characterization for Therapeutic Applications, and Challenges for Clinical Translation. ACS Nano.

[62] Wu P., Jiang X., Yin S., Yang Y., Liu T., Wang K. (2021). Biomimetic recombinant of red blood cell membranes for improved photothermal therapy. J. Nanobiotechnol..

[63] Quach A., Ferrante A. (2017). The Application of Dextran Sedimentation as an Initial Step in Neutrophil Purification Promotes Their Stimulation, due to the Presence of Monocytes. J. Immunol. Res..

[64] Wang C., Wu S. (2022). Research update on cell membrane camouflaged nanoparticles for cancer therapy. Front. Bioeng. Biotechnol..

[65] Zhang Y., Chen Q., Zhu Y., Pei M., Wang K., Qu X., Zhang Y., Gao J., Qin H. (2022). Targeting inorganic nanoparticles to tumors using biological membrane-coated technology. MedComm.

[66] Li Y., Sun H., Cao D., Guo Y., Wu D., Yang M., Wang H., Shao X., Li Y., Liang Y. (2025). Overcoming Biological Barriers in Cancer Therapy: Cell Membrane-Based Nanocarrier Strategies for Precision Delivery. Int. J. Nanomed..

[67] Guido C., Maiorano G., Cortese B., D’Amone S., Palamà I.E. (2020). Biomimetic Nanocarriers for Cancer Target Therapy. Bioengineering.

[68] Wu H., Zhang T., Li N., Gao J. (2023). Cell membrane-based biomimetic vehicles for effective central nervous system target delivery: Insights and challenges. J. Control. Release.

[69] Buzas E.I. (2023). The roles of extracellular vesicles in the immune system. Nat. Rev. Immunol..

[70] Kumar M.A., Baba S.K., Sadida H.Q., Marzooqi S.A., Jerobin J., Altemani F.H., Algehainy N., Alanazi M.A., Abou-Samra A.B., Kumar R. (2024). Extracellular vesicles as tools and targets in therapy for diseases. Signal Transduct. Target. Ther..

[71] Luo R., Liu M., Tan T., Yang Q., Wang Y., Men L., Zhao L., Zhang H., Wang S., Xie T. (2021). Emerging Significance and Therapeutic Potential of Extracellular vesicles. Int. J. Biol. Sci..

[72] Wang Z., Zhou X., Kong Q., He H., Sun J., Qiu W., Zhang L., Yang M. (2024). Extracellular Vesicle Preparation and Analysis: A State-of-the-Art Review. Adv. Sci..

[73] Sun D., Ma Y., Wu M., Chen Z., Zhang L., Lu J. (2023). Recent progress in aptamer-based microfluidics for the detection of circulating tumor cells and extracellular vesicles. J. Pharm. Anal..

[74] Zeng M., Liu M., Tao X., Yin X., Shen C., Wang X. (2024). Emerging Trends in the Application of Extracellular Vesicles as Novel Oral Delivery Vehicles for Therapeutics in Inflammatory Diseases. Int. J. Nanomed..

[75] Tong L., Zhang S., Huang R., Yi H., Wang J.W. (2022). Extracellular vesicles as a novel photosensitive drug delivery system for enhanced photodynamic therapy. Front. Bioeng. Biotechnol..

[76] Su X., Wang H., Li Q., Chen Z. (2025). Extracellular Vesicles: A Review of Their Therapeutic Potentials, Sources, Biodistribution, and Administration Routes. Int. J. Nanomed..

[77] Ma S.R., Xia H.F., Gong P., Yu Z.L. (2023). Red Blood Cell-Derived Extracellular Vesicles: An Overview of Current Research Progress, Challenges, and Opportunities. Biomedicines.

[78] Zhang Y., Sun C., Wang C., Jankovic K.E., Dong Y. (2021). Lipids and Lipid Derivatives for RNA Delivery. Chem. Rev..

[79] Yang L., Huang S., Zhang Z., Liu Z., Zhang L. (2022). Roles and Applications of Red Blood Cell-Derived Extracellular Vesicles in Health and Diseases. Int. J. Mol. Sci..

[80] Lam B.W.S., Tan M., Gao C., Pham T.T., Tran L.T.N., Nguyen L.N., Sidik H., Lim M.B.H., Le A.H., Nguyen T.T.T. (2025). Extracellular Vesicles Administered via Intrathecal Injection Mediate Safe Delivery of Nucleic Acids to the Central Nervous System for Gene Therapy. J. Extracell. Vesicles.

[81] Shao X., Yan C., Wang C., Wang C., Cao Y., Zhou Y., Guan P., Hu X., Zhu W., Ding S. (2022). Advanced nanomaterials for modulating Alzheimer’s related amyloid aggregation. Nanoscale Adv..

[82] Salybekov A.A., Kunikeyev A.D., Kobayashi S., Asahara T. (2021). Latest Advances in Endothelial Progenitor Cell-Derived Extracellular Vesicles Translation to the Clinic. Front. Cardiovasc. Med..

[83] Pham T.T., Le A.H., Dang C.P., Chong S.Y., Do D.V., Peng B., Jayasinghe M.K., Ong H.B., Hoang D.V., Louise R.A. (2023). Endocytosis of red blood cell extracellular vesicles by macrophages leads to cytoplasmic heme release and prevents foam cell formation in atherosclerosis. J. Extracell. Vesicles.

[84] Dong X., Dong J.F., Zhang J. (2023). Roles and therapeutic potential of different extracellular vesicle subtypes on traumatic brain injury. Cell Commun. Signal.

[85] Liu L., Deng Q.J. (2022). Role of platelet-derived extracellular vesicles in traumatic brain injury-induced coagulopathy and inflammation. Neural Regen. Res..

[86] Corvigno S., Johnson A.M., Wong K.K., Cho M.S., Afshar-Kharghan V., Menter D.G., Sood A.K. (2022). Novel Markers for Liquid Biopsies in Cancer Management: Circulating Platelets and Extracellular Vesicles. Mol. Cancer Ther..

[87] Nyam-Erdene A., Nebie O., Delila L., Buée L., Devos D., Chou S.Y., Blum D., Burnouf T. (2021). Characterization and Chromatographic Isolation of Platelet Extracellular Vesicles from Human Platelet Lysates for Applications in Neuroregenerative Medicine. ACS Biomater. Sci. Eng..

[88] Wang Y., Zhao M., Liu S., Guo J., Lu Y., Cheng J., Liu J. (2020). Macrophage-derived extracellular vesicles: Diverse mediators of pathology and therapeutics in multiple diseases. Cell Death Dis..

[89] Zhang C., Li D., Hu H., Wang Z., An J., Gao Z., Zhang K., Mei X., Wu C., Tian H. (2021). Engineered extracellular vesicles derived from primary M2 macrophages with anti-inflammatory and neuroprotective properties for the treatment of spinal cord injury. J. Nanobiotechnol..

[90] Song Y., Hu J., Ma C., Liu H., Li Z., Yang Y. (2024). Macrophage-Derived Exosomes as Advanced Therapeutics for Inflammation: Current Progress and Future Perspectives. Int. J. Nanomed..

[91] Jung I., Shin S., Baek M.C., Yea K. (2024). Modification of immune cell-derived exosomes for enhanced cancer immunotherapy: Current advances and therapeutic applications. Exp. Mol. Med..

[92] Youn Y.J., Shrestha S., Lee Y.B., Kim J.K., Lee J.H., Hur K., Mali N.M., Nam S.W., Kim S.H., Lee S. (2021). Neutrophil-derived trail is a proinflammatory subtype of neutrophil-derived extracellular vesicles. Theranostics.

[93] Zou Y., Liao L., Dai J., Mazhar M., Yang G., Wang H., Dechsupa N., Wang L. (2023). Mesenchymal stem cell-derived extracellular vesicles/exosome: A promising therapeutic strategy for intracerebral hemorrhage. Regen. Ther..

[94] Go V., Sarikaya D., Zhou Y., Bowley B.G.E., Pessina M.A., Rosene D.L., Zhang Z.G., Chopp M., Finklestein S.P., Medalla M. (2021). Extracellular vesicles derived from bone marrow mesenchymal stem cells enhance myelin maintenance after cortical injury in aged rhesus monkeys. Exp. Neurol..

[95] Yu T., Xu Q., Chen X., Deng X., Chen N., Kou M.T., Huang Y., Guo J., Xiao Z., Wang J. (2024). Biomimetic nanomaterials in myocardial infarction treatment: Harnessing bionic strategies for advanced therapeutics. Mater. Today Bio.

[96] Hua Z., Zhou N., Zhou Z., Fu Z., Guo R., Akogo H.Y., Yang J., Yu M., Jiang Y., Lan S. (2025). Intranasal administration of stem cell derivatives for the treatment of AD animal models: A systematic review and meta-analysis. Stem Cell Res. Ther..

[97] Calvo B., Schembri-Wismayer P., Durán-Alonso M.B. (2025). Age-Related Neurodegenerative Diseases: A Stem Cell’s Perspective. Cells.

[98] Ding M., Shen Y., Wang P., Xie Z., Xu S., Zhu Z., Wang Y., Lyu Y., Wang D., Xu L. (2018). Exosomes Isolated From Human Umbilical Cord Mesenchymal Stem Cells Alleviate Neuroinflammation and Reduce Amyloid-Beta Deposition by Modulating Microglial Activation in Alzheimer’s Disease. Neurochem. Res..

[99] Kim K.M., Meng Q., Perez de Acha O., Mustapic M., Cheng A., Eren E., Kundu G., Piao Y., Munk R., Wood W.H. (2020). Mitochondrial RNA in Alzheimer’s Disease Circulating Extracellular Vesicles. Front. Cell Dev. Biol..

[100] Corbeil D., Santos M.F., Karbanová J., Kurth T., Rappa G., Lorico A. (2020). Uptake and Fate of Extracellular Membrane Vesicles: Nucleoplasmic Reticulum-Associated Late Endosomes as a New Gate to Intercellular Communication. Cells.

[101] Małys M.S., Aigner C., Schulz S.M., Schachner H., Rees A.J., Kain R. (2021). Isolation of Small Extracellular Vesicles from Human Sera. Int. J. Mol. Sci..

[102] Li M., Fang F., Sun M., Zhang Y., Hu M., Zhang J. (2022). Extracellular vesicles as bioactive nanotherapeutics: An emerging paradigm for regenerative medicine. Theranostics.

[103] Shpigelman J., Lao F.S., Yao S., Li C., Saito T., Sato-Kaneko F., Nolan J.P., Shukla N.M., Pu M., Messer K. (2021). Generation and Application of a Reporter Cell Line for the Quantitative Screen of Extracellular Vesicle Release. Front. Pharmacol..

[104] Mentkowski K.I., Snitzer J.D., Rusnak S., Lang J.K. (2018). Therapeutic Potential of Engineered Extracellular Vesicles. AAPS J..

[105] Bian S., Liu H. (2019). Isolation and identification methods of extracellular vesicles. J. N. Med..

[106] Tran H.L., Zheng W., Issadore D.A., Im H., Cho Y.K., Zhang Y., Liu D., Liu Y., Li B., Liu F. (2025). Extracellular Vesicles for Clinical Diagnostics: From Bulk Measurements to Single-Vesicle Analysis. ACS Nano.

[107] Ning K., Zou W., Xu P., Cheng F., Zhang E.Y., Zhang-Chen A., Kleiboeker S., Qiu J. (2023). Identification of AXL as a co-receptor for human parvovirus B19 infection of human erythroid progenitors. Sci. Adv..

[108] Thangaraju K., Neerukonda S.N., Katneni U., Buehler P.W. (2020). Extracellular Vesicles from Red Blood Cells and Their Evolving Roles in Health, Coagulopathy and Therapy. Int. J. Mol. Sci..

[109] Liu J., Shen T., Zhang Y., Wei X., Bao Y., Ai R., Gan S., Wang D., Lai X., Zhao L. (2024). Cell dehydration enables massive production of engineered membrane vesicles with therapeutic functions. J. Extracell. Vesicles.

[110] Sun B., Sawant H., Borthakur A., Bihl J.C. (2023). Emerging therapeutic role of gut microbial extracellular vesicles in neurological disorders. Front. Neurosci..

[111] Bashir Y., Khan A.U. (2022). The interplay between the gut-brain axis and the microbiome: A perspective on psychiatric and neurodegenerative disorders. Front. Neurosci..

[112] Cheung K.C.P., Jiao M., Xingxuan C., Wei J. (2023). Extracellular vesicles derived from host and gut microbiota as promising nanocarriers for targeted therapy in osteoporosis and osteoarthritis. Front. Pharmacol..

[113] Guo J., Huang Z., Wang Q., Wang M., Ming Y., Chen W., Huang Y., Tang Z., Huang M., Liu H. (2025). Opportunities and challenges of bacterial extracellular vesicles in regenerative medicine. J. Nanobiotechnol..

[114] Nie X., Li Q., Chen X., Onyango S., Xie J., Nie S. (2024). Bacterial extracellular vesicles: Vital contributors to physiology from bacteria to host. Microbiol. Res..

[115] Dai K., Liao B., Huang X., Liu Q. (2025). Consistency in bacterial extracellular vesicle production: Key to their application in human health. Extracell. Vesicles Circ. Nucleic Acids.

[116] Sandanusova M., Turkova K., Pechackova E., Kotoucek J., Roudnicky P., Sindelar M., Kubala L., Ambrozova G. (2024). Growth phase matters: Boosting immunity via Lacticasebacillus-derived membrane vesicles and their interactions with TLR2 pathways. J. Extracell. Biol..

[117] Mantella V., Bienz S., Brigger F., Baulier E., Ramus M., Zoratto N., Honrath S., Naresh K., Sander S., Dengjel J. (2025). Isolation of bacterial extracellular vesicles from raw samples using a portable microstructured electrochemical device. Drug Deliv. Transl. Res..

[118] Wang S., Luo J., Wang H., Chen T., Sun J., Xi Q., Zhang Y. (2024). Extracellular Vesicles: A Crucial Player in the Intestinal Microenvironment and Beyond. Int. J. Mol. Sci..

[119] Wang Y., Luo X., Xiang X., Hao C., Ma D. (2023). Roles of bacterial extracellular vesicles in systemic diseases. Front. Microbiol..

[120] Zhu Z., Antenucci F., Villumsen K.R., Bojesen A.M. (2021). Bacterial Outer Membrane Vesicles as a Versatile Tool in Vaccine Research and the Fight against Antimicrobial Resistance. mBio.

[121] Jiang B., Huang J. (2024). Influences of bacterial extracellular vesicles on macrophage immune functions. Front. Cell. Infect. Microbiol..

[122] Xie J., Haesebrouck F., Van Hoecke L., Vandenbroucke R.E. (2023). Bacterial extracellular vesicles: An emerging avenue to tackle diseases. Trends Microbiol..

[123] Xie J., Li Q., Haesebrouck F., Van Hoecke L., Vandenbroucke R.E. (2022). The tremendous biomedical potential of bacterial extracellular vesicles. Trends Biotechnol..

[124] De Langhe N., Van Dorpe S., Guilbert N., Vander Cruyssen A., Roux Q., Deville S., Dedeyne S., Tummers P., Denys H., Vandekerckhove L. (2024). Mapping bacterial extracellular vesicle research: Insights, best practices and knowledge gaps. Nat. Commun..

[125] Chronopoulos A., Kalluri R. (2020). Emerging role of bacterial extracellular vesicles in cancer. Oncogene.

[126] Ho M.Y., Liu S., Xing B. (2024). Bacteria extracellular vesicle as nanopharmaceuticals for versatile biomedical potential. Nano Converg..

[127] Liu Y., Zou Y., Feng C., Lee A., Yin J., Chung R., Park J.B., Rizos H., Tao W., Zheng M. (2020). Charge Conversional Biomimetic Nanocomplexes as a Multifunctional Platform for Boosting Orthotopic Glioblastoma RNAi Therapy. Nano Lett..

[128] Yin T., Fan Q., Hu F., Ma X., Yin Y., Wang B., Kuang L., Hu X., Xu B., Wang Y. (2022). Engineered Macrophage-Membrane-Coated Nanoparticles with Enhanced PD-1 Expression Induce Immunomodulation for a Synergistic and Targeted Antiglioblastoma Activity. Nano Lett..

[129] Wang X., Ding H., Li Z., Peng Y., Tan H., Wang C., Huang G., Li W., Ma G., Wei W. (2022). Exploration and functionalization of M1-macrophage extracellular vesicles for effective accumulation in glioblastoma and strong synergistic therapeutic effects. Signal Transduct. Target. Ther..

[130] You H., Zhang S., Zhang Y., Chen Q., Wu Y., Zhou Z., Zhao Z., Su B., Li X., Guo Y. (2025). Engineered Bacterial Outer Membrane Vesicles-Based Doxorubicin and CD47-siRNA Co-Delivery Nanoplatform Overcomes Immune Resistance to Potentiate the Immunotherapy of Glioblastoma. Adv. Mater..

[131] Lv W., Xu J., Wang X., Li X., Xu Q., Xin H. (2018). Bioengineered Boronic Ester Modified Dextran Polymer Nanoparticles as Reactive Oxygen Species Responsive Nanocarrier for Ischemic Stroke Treatment. ACS Nano.

[132] Quan X., Han Y., Lu P., Ding Y., Wang Q., Li Y., Wei J., Huang Q., Wang R., Zhao Y. (2022). Annexin V-Modified Platelet-Biomimetic Nanomedicine for Targeted Therapy of Acute Ischemic Stroke. Adv. Healthc. Mater..

[133] Dong Z., Tang L., Zhang Y., Ma X., Yin Y., Kuang L., Fan Q., Wang B., Hu X., Yin T. (2024). A Homing Peptide Modified Neutrophil Membrane Biomimetic Nanoparticles in Response to ROS/inflammatory Microenvironment for Precise Targeting Treatment of Ischemic Stroke. Adv. Funct. Mater..

[134] Pan J., Wang Z., Huang X., Xue J., Zhang S., Guo X., Zhou S. (2023). Bacteria-Derived Outer-Membrane Vesicles Hitchhike Neutrophils to Enhance Ischemic Stroke Therapy. Adv. Mater..

[135] Yang Z., Gao Z., Yang Z., Zhang Y., Chen H., Yang X., Fang X., Zhu Y., Zhang J., Ouyang F. (2022). *Lactobacillus* plantarum-derived extracellular vesicles protect against ischemic brain injury via the microRNA-101a-3p/c-Fos/TGF-β axis. Pharmacol. Res..

[136] Cheng M., Ye C., Tian C., Zhao D., Li H., Sun Z., Miao Y., Zhang Q., Wang J., Dou Y. (2023). Engineered macrophage-biomimetic versatile nanoantidotes for inflammation-targeted therapy against Alzheimer?s disease by neurotoxin neutralization and immune recognition suppression. Bioact. Mater..

[137] Liu C., Zhang W., Zhang H., Zhao C., Du X., Ren J., Qu X. (2024). Biomimetic engineering of a neuroinflammation-targeted MOF nanozyme scaffolded with photo-trigger released CO for the treatment of Alzheimer’s disease. Chem. Sci..

[138] Reza-Zaldivar E.E., Hernández-Sapiéns M.A., Gutiérrez-Mercado Y.K., Sandoval-Ávila S., Gomez-Pinedo U., Márquez-Aguirre A.L., Vázquez-Méndez E., Padilla-Camberos E., Canales-Aguirre A.A. (2019). Mesenchymal stem cell-derived exosomes promote neurogenesis and cognitive function recovery in a mouse model of Alzheimer’s disease. Neural Regen. Res..

[139] Li S., Ren Y., Xiao X., Chen Y., Zhao W., Liu H., Ding Z., Xu Y., Xin H., Guo Y. (2025). Enhanced neuroprotection in Parkinson’s disease by neutrophil-biomimetic nanovesicles through autophagy inhibition and apoptosis antagonism. Chem. Eng. J..

[140] Pouyan A., Ghorbanlo M., Eslami M., Jahanshahi M., Ziaei E., Salami A., Mokhtari K., Shahpasand K., Farahani N., Meybodi T.E. (2025). Glioblastoma multiforme: Insights into pathogenesis, key signaling pathways, and therapeutic strategies. Mol. Cancer.

[141] Liu J., Yang F., Hu J., Zhang X. (2024). Nanoparticles for efficient drug delivery and drug resistance in glioma: New perspectives. CNS Neurosci. Ther..

[142] Yuan B., Wang G., Tang X., Tong A., Zhou L. (2022). Immunotherapy of glioblastoma: Recent advances and future prospects. Hum. Vaccines Immunother..

[143] Qin C., Yang S., Chu Y.H., Zhang H., Pang X.W., Chen L., Zhou L.Q., Chen M., Tian D.S., Wang W. (2022). Signaling pathways involved in ischemic stroke: Molecular mechanisms and therapeutic interventions. Signal Transduct. Target. Ther..

[144] Ajoolabady A., Wang S., Kroemer G., Penninger J.M., Uversky V.N., Pratico D., Henninger N., Reiter R.J., Bruno A., Joshipura K. (2021). Targeting autophagy in ischemic stroke: From molecular mechanisms to clinical therapeutics. Pharmacol. Ther..

[145] Mendelson S.J., Prabhakaran S. (2021). Diagnosis and Management of Transient Ischemic Attack and Acute Ischemic Stroke: A Review. Jama.

[146] Fukuta T., Oku N., Kogure K. (2022). Application and Utility of Liposomal Neuroprotective Agents and Biomimetic Nanoparticles for the Treatment of Ischemic Stroke. Pharmaceutics.

[147] Ji P., Xu Q., Li J., Wang Z., Mao W., Yan P. (2024). Advances in nanoparticle-based therapeutics for ischemic stroke: Enhancing drug delivery and efficacy. Biomed. Pharmacother..

[148] Migliavacca M., Correa-Paz C., Pérez-Mato M., Bielawski P.B., Zhang I., Marie P., Hervella P., Rubio M., Maysinger D., Vivien D. (2024). Thrombolytic therapy based on lyophilized platelet-derived nanocarriers for ischemic stroke. J. Nanobiotechnol..

[149] Ruan H., Li Y., Zheng D., Deng L., Chen G., Zhang X., Tang Y., Cui W. (2023). Engineered extracellular vesicles for ischemic stroke treatment. Innovation.

[150] Breijyeh Z., Karaman R. (2020). Comprehensive Review on Alzheimer’s Disease: Causes and Treatment. Molecules.

[151] Ferrari C., Sorbi S. (2021). The complexity of Alzheimer’s disease: An evolving puzzle. Physiol. Rev..

[152] Alcolea D., Beeri M.S., Rojas J.C., Gardner R.C., Lleó A. (2023). Blood Biomarkers in Neurodegenerative Diseases: Implications for the Clinical Neurologist. Neurology.

[153] Twarowski B., Herbet M. (2023). Inflammatory Processes in Alzheimer’s Disease—Pathomechanism, Diagnosis and Treatment: A Review. Int. J. Mol. Sci..

[154] Ye Y., Gao M., Shi W., Gao Y., Li Y., Yang W., Zheng X., Lu X. (2024). The immunomodulatory effects of mesenchymal stem cell-derived extracellular vesicles in Alzheimer’s disease. Front. Immunol..

[155] Morris H.R., Spillantini M.G., Sue C.M., Williams-Gray C.H. (2024). The pathogenesis of Parkinson’s disease. Lancet.

[156] Tolosa E., Garrido A., Scholz S.W., Poewe W. (2021). Challenges in the diagnosis of Parkinson’s disease. Lancet Neurol..

[157] Vijiaratnam N., Simuni T., Bandmann O., Morris H.R., Foltynie T. (2021). Progress towards therapies for disease modification in Parkinson’s disease. Lancet Neurol..

[158] Gattoni M.F., Gobbo S., Feroldi S., Salvatore A., Navarro J., Sorbi S., Saibene F.L. (2025). Identification of Cognitive Training for Individuals with Parkinson’s Disease: A Systematic Review. Brain Sci..

[159] Izco M., Blesa J., Schleef M., Schmeer M., Porcari R., Al-Shawi R., Ellmerich S., de Toro M., Gardiner C., Seow Y. (2019). Systemic Exosomal Delivery of shRNA Minicircles Prevents Parkinsonian Pathology. Mol. Ther..

[160] Sun K., Zheng X., Jin H., Yu F., Zhao W. (2022). Exosomes as CNS Drug Delivery Tools and Their Applications. Pharmaceutics.

[161] Wang P., Lan G., Xu B., Yu Z., Tian C., Lei X., Meissner W.G., Feng T., Yang Y., Zhang J. (2023). α-Synuclein-carrying astrocytic extracellular vesicles in Parkinson pathogenesis and diagnosis. Transl. Neurodegener..

[162] Hou J.J., Li W.W., Wang X.L., Ma A.H., Qin Y.H. (2023). Efficacy of extracellular vesicles as a cell-free therapy in colitis: A systematic review and meta-analysis of animal studies. Front. Pharmacol..

[163] Lee D., Minko T. (2021). Nanotherapeutics for Nose-to-Brain Drug Delivery: An Approach to Bypass the Blood Brain Barrier. Pharmaceutics.

[164] Kisku A., Nishad A., Agrawal S., Paliwal R., Datusalia A.K., Gupta G., Singh S.K., Dua K., Sulakhiya K. (2024). Recent developments in intranasal drug delivery of nanomedicines for the treatment of neuropsychiatric disorders. Front. Med..

[165] Marcello E., Chiono V. (2023). Biomaterials-Enhanced Intranasal Delivery of Drugs as a Direct Route for Brain Targeting. Int. J. Mol. Sci..

[166] Costa C.P., Moreira J.N., Sousa Lobo J.M., Silva A.C. (2021). Intranasal delivery of nanostructured lipid carriers, solid lipid nanoparticles and nanoemulsions: A current overview of in vivo studies. Acta Pharm. Sin. B.

[167] Huang Q., Chen X., Yu S., Gong G., Shu H. (2024). Research progress in brain-targeted nasal drug delivery. Front. Aging Neurosci..

[168] Georgiopoulos C., Witt S.T., Haller S., Dizdar N., Zachrisson H., Engström M., Larsson E.M. (2019). A study of neural activity and functional connectivity within the olfactory brain network in Parkinson’s disease. Neuroimage Clin..

[169] Tapia-Arellano A., Cabrera P., Cortés-Adasme E., Riveros A., Hassan N., Kogan M.J. (2024). Tau- and α-synuclein-targeted gold nanoparticles: Applications, opportunities, and future outlooks in the diagnosis and therapy of neurodegenerative diseases. J. Nanobiotechnol..

[170] Chen Y., Zhang C., Huang Y., Ma Y., Song Q., Chen H., Jiang G., Gao X. (2024). Intranasal drug delivery: The interaction between nanoparticles and the nose-to-brain pathway. Adv. Drug Deliv. Rev..

[171] Crowe T.P., Greenlee M.H.W., Kanthasamy A.G., Hsu W.H. (2018). Mechanism of intranasal drug delivery directly to the brain. Life Sci..

[172] Selvaraj K., Gowthamarajan K., Karri V.V.S.R. (2018). Nose to brain transport pathways an overview: Potential of nanostructured lipid carriers in nose to brain targeting. Artif. Cells Nanomed. Biotechnol..

[173] Rassu G., Sorrenti M., Catenacci L., Pavan B., Ferraro L., Gavini E., Bonferoni M.C., Giunchedi P., Dalpiaz A. (2021). Versatile Nasal Application of Cyclodextrins: Excipients and/or Actives?. Pharmaceutics.

[174] Dalaqua M., do Nascimento F.B.P., Miura L.K., Reis F., Garcia M.R.T., Barbosa Júnior A.A. (2021). Magnetic resonance imaging of the cranial nerves in congenital, traumatic, and vascular diseases: A pictorial essay. Radiol. Bras..

[175] Khan A.R., Liu M., Khan M.W., Zhai G. (2017). Progress in brain targeting drug delivery system by nasal route. J. Control. Release.

[176] Ferreira M.D., Duarte J., Veiga F., Paiva-Santos A.C., Pires P.C. (2023). Nanosystems for Brain Targeting of Antipsychotic Drugs: An Update on the Most Promising Nanocarriers for Increased Bioavailability and Therapeutic Efficacy. Pharmaceutics.

[177] Pan J., Fu Y., Yang P., Li W., Luo Z., Zhang A., Du J., Mei F., Liu F., Qi S. (2025). The Cerebral Lymphatic System: Function, Controversies, and Therapeutic Approaches for Central Nervous System Diseases. Cell. Mol. Neurobiol..

[178] Xu J., Tao J., Wang J. (2020). Design and Application in Delivery System of Intranasal Antidepressants. Front. Bioeng. Biotechnol..

[179] Maher R., Moreno-Borrallo A., Jindal D., Mai B.T., Ruiz-Hernandez E., Harkin A. (2023). Intranasal Polymeric and Lipid-Based Nanocarriers for CNS Drug Delivery. Pharmaceutics.

[180] Chung S., Peters J.M., Detyniecki K., Tatum W., Rabinowicz A.L., Carrazana E. (2023). The nose has it: Opportunities and challenges for intranasal drug administration for neurologic conditions including seizure clusters. Epilepsy Behav. Rep..

[181] Liu T., Wang Y., Zhang M., Zhang J., Kang N., Zheng L., Ding Z. (2024). The Optimization Design of Macrophage Membrane Camouflaging Liposomes for Alleviating Ischemic Stroke Injury through Intranasal Delivery. Int. J. Mol. Sci..

[182] Liu T., Zhang M., Zhang J., Kang N., Zheng L., Ding Z. (2024). Targeted Delivery of Macrophage Membrane Biomimetic Liposomes Through Intranasal Administration for Treatment of Ischemic Stroke. Int. J. Nanomed..

[183] Borlongan C.V., Lee J.Y., D’Egidio F., de Kalbermatten M., Garitaonandia I., Guzman R. (2024). Nose-to-brain delivery of stem cells in stroke: The role of extracellular vesicles. Stem Cells Transl. Med..

[184] Sha S., Sun C., Gao X., Bi W., Chen H., Ren W., Wang L., Luo E., Xing C., Du H. (2025). Engineered Stem Cell Membrane-Coated Nanodrugs for Targeted Therapy of Alzheimer’s Disease. ACS Appl. Mater. Interfaces.

[185] Xie X., Song Q., Dai C., Cui S., Tang R., Li S., Chang J., Li P., Wang J., Li J. (2023). Clinical safety and efficacy of allogenic human adipose mesenchymal stromal cells-derived exosomes in patients with mild to moderate Alzheimer’s disease: A phase I/II clinical trial. Gen. Psychiatry.

[186] Madhu L.N., Kodali M., Upadhya R., Rao S., Somayaji Y., Attaluri S., Shuai B., Kirmani M., Gupta S., Maness N. (2024). Extracellular vesicles from human-induced pluripotent stem cell-derived neural stem cells alleviate proinflammatory cascades within disease-associated microglia in Alzheimer’s disease. J. Extracell. Vesicles.

[187] Saha P., Kathuria H., Pandey M.M. (2023). Intranasal nanotherapeutics for brain targeting and clinical studies in Parkinson’s disease. J. Control. Release.

[188] Narbute K., Piļipenko V., Pupure J., Dzirkale Z., Jonavičė U., Tunaitis V., Kriaučiūnaitė K., Jarmalavičiūtė A., Jansone B., Kluša V. (2019). Intranasal Administration of Extracellular Vesicles Derived from Human Teeth Stem Cells Improves Motor Symptoms and Normalizes Tyrosine Hydroxylase Expression in the Substantia Nigra and Striatum of the 6-Hydroxydopamine-Treated Rats. Stem Cells Transl. Med..

[189] Haney M.J., Klyachko N.L., Zhao Y., Gupta R., Plotnikova E.G., He Z., Patel T., Piroyan A., Sokolsky M., Kabanov A.V. (2015). Exosomes as drug delivery vehicles for Parkinson’s disease therapy. J. Control. Release.

[190] Ferreira N.N., Leite C.M., Moreno N.S., Miranda R.R., Pincela Lins P.M., Rodero C.F., de Oliveira Junior E., Lima E.M., Reis R.M., Zucolotto V. (2025). Nose-to-Brain Delivery of Biomimetic Nanoparticles for Glioblastoma Targeted Therapy. ACS Appl. Mater. Interfaces.

[191] Shen H., Aggarwal N., Cui B., Foo G.W., He Y., Srivastava S.K., Li S., Seah M.Z.X., Wun K.S., Ling H. (2025). Engineered commensals for targeted nose-to-brain drug delivery. Cell.

[192] Zhou Y., Wang X., Zhang D., Cui H., Tian X., Du W., Yang Z., Wan D., Qiu Z., Liu C. (2025). Precision-Guided Stealth Missiles in Biomedicine: Biological Carrier-Mediated Nanomedicine Hitchhiking Strategy. Adv. Sci..

[193] Dong X., Gao J., Su Y., Wang Z. (2020). Nanomedicine for Ischemic Stroke. Int. J. Mol. Sci..

[194] Sun Y., Sun F., Xu W., Qian H. (2023). Engineered Extracellular Vesicles as a Targeted Delivery Platform for Precision Therapy. Tissue Eng. Regen. Med..

[195] Dave K.M., Pinky P.P., Manickam D.S. (2025). Molecular engineering of extracellular vesicles for drug delivery: Strategies, challenges, and perspectives. J. Control. Release.

[196] Liu H., Zhang H., Han Y., Hu Y., Geng Z., Su J. (2022). Bacterial extracellular vesicles-based therapeutic strategies for bone and soft tissue tumors therapy. Theranostics.

[197] Fan Y., Chen M., Zhang J., Maincent P., Xia X., Wu W. (2018). Updated Progress of Nanocarrier-Based Intranasal Drug Delivery Systems for Treatment of Brain Diseases. Crit. Rev. Ther. Drug Carr. Syst..

[198] Yang T., Zhai J., Hu D., Yang R., Wang G., Li Y., Liang G. (2022). “Targeting Design” of Nanoparticles in Tumor Therapy. Pharmaceutics.

[199] Liu B.D., Akbar R., Oliverio A., Thapa K., Wang X., Fan G.C. (2024). Bacterial Extracellular Vesicles in the Regulation of Inflammatory Response and Host-Microbe Interactions. Shock.

[200] Piunti C., Cimetta E. (2023). Microfluidic approaches for producing lipid-based nanoparticles for drug delivery applications. Biophys. Rev..

